# Untreated HIV-1 infection and low CD4^+^ T cell counts and their effect on endemic human coronavirus (re)infection

**DOI:** 10.1371/journal.pgph.0004610

**Published:** 2025-06-18

**Authors:** Ferdyansyah Sechan, Anne W. M. van den Hurk, T. Sonia Boender, Maria Prins, Amy Matser, Margreet Bakker, Neeltje A. Kootstra, Lia van der Hoek

**Affiliations:** 1 Laboratory of Experimental Virology, Department of Medical Microbiology and Infection Prevention, Amsterdam UMC, Location AMC, University of Amsterdam, Amsterdam, the Netherlands; 2 Amsterdam institute for Immunology and Infectious diseases, Amsterdam, The Netherlands; 3 Department of Internal and Experimental Vascular Medicine, Amsterdam UMC, Location AMC, University of Amsterdam, Amsterdam, the Netherlands; 4 Amsterdam Gastroenterology, Endocrinology & Metabolism, Amsterdam UMC, Amsterdam, the Netherlands; 5 Amsterdam Cardiovascular Sciences, Diabetes & Metabolism, Amsterdam UMC, Amsterdam, the Netherlands; 6 Department of Infectious Diseases, Public Health Service Amsterdam, Amsterdam, Netherlands; 7 Department of Infectious Diseases, Amsterdam UMC, Location AMC, University of Amsterdam, Amsterdam, the Netherlands; 8 Department of Health Sciences, Faculty of Science, Vrije Universiteit (VU) Amsterdam, Amsterdam, Netherlands; 9 Amsterdam Public Health Research Institute Amsterdam, Amsterdam, Netherlands; 10 Laboratory for Viral Immune Pathogenesis, Department of Experimental Immunology, Amsterdam UMC, location AMC, University of Amsterdam, Amsterdam, Netherlands; University of California Irvine, UNITED STATES OF AMERICA

## Abstract

People living with HIV-1 (PLWH) treated with combination antiretroviral therapy (cART) have similar incidence of SARS-CoV-2 infection compared to people without HIV-1 (PWoH). Yet, roughly 25% PLWH worldwide are currently not accessing cART. The influence of CD4^+^ T cell depletion on human coronavirus (HCoV) (re)infection risk, including SARS-CoV-2, is largely unknown. In this research, we investigated the incidence of infection by the four endemic HCoVs (HCoV-NL63, HCoV-229E, HCoV-OC43, and HCoV-HKU1), to inform on future reinfections by SARS-CoV-2. We compared the HCoV infection incidence rate between PLWH (*n* = 24) and PWoH (*n* = 25) who were followed up in 1984–1993; i.e., before cART became generally available in high income countries. Both populations were followed up at 6-month intervals for 7 or 8 years. We also compared the HCoV infection incidence rate among PLWH with and without immune deficiency, defined as CD4^+^ T cell count < 350 cell/mm^3^ and > 350 cell/mm^3^ respectively. We found that the antibody levels for all HCoVs were significantly lower in PLWH than PWoH across all timepoints. However, we observed no significant difference on HCoV infection incidence rate between PLWH and PWoH. We also observed no difference in HCoV infection incidence rate among PLWH with and without immune deficiency. We conclude that PLWH not on cART may not be at increased risk of HCoV reinfections.

## 1. Introduction

At the start of the Coronavirus 2019 (COVID-19) pandemic, human immunodeficiency virus 1 (HIV-1) infection was considered a risk factor for SARS-CoV-2 acquisition [1]. Yet, it is now accepted that people on combination antiretroviral therapy (cART) with well-controlled HIV-1 infection have similar incidence of SARS-CoV-2 infection compared to the HIV-1 seronegative population [2,3]. The recovery of CD4+ T cell count as the result of HIV-1 suppression via cART is assumed to be the reason behind the similar risk of SARS-CoV-2 infection between people living with HIV-1 (PLWH) and people without HIV-1 (PWoH) [3,4]. However, despite the ever-expanding coverage of cART, it is estimated in 2021 that around 25% of PLWH worldwide were not accessing cART due to various reasons [5]. In the light of current SARS-CoV-2 endemicity and potential reinfections, the influence of CD4+ T cell depletion on PLWH not on cART towards the vulnerability of SARS-CoV-2 (re)infections needs to be investigated.

Human coronavirus (HCoV) NL63, HCoV-229E, HCoV-OC43, and HCoV-HKU1 are collectively known as endemic, or seasonal, HCoVs, due to them causing seasonal common-cold-like illness in humans. They have been infecting humans for centuries [6,7], and first infection (and seroconversion) by these HCoVs occurs during the first years of life [8,9]. There are similarities between endemic HCoVs and SARS-CoV-2, making endemic HCoVs a suitable model to study SARS-CoV-2 (re)infection vulnerability. First of all, they are all coronaviruses, secondly, both SARS-CoV-2 and HCoV-NL63 utilize ACE2 for cellular entry during infection [10,11], and thirdly, SARS-CoV-2 belongs to the same genus as HCoV-OC43 and HCoV-HKU1 (genus *Betacoronaviruses*) [11,12].

Endemic coronaviruses, from human or other animals, often reinfect their host (reviewed in [13]). In human volunteers, experimental reinfection by a HCoV - one year post previous infection - was successful when antibodies recognizing the virus had waned to pre-first-infection levels [14,15], a strong indication that protective immunity decreases with time. Next to that, reinfections are pushed by evolution of HCoVs [16]. Since experimental infection studies cannot be done in PLWH not on cART, we aim to study vulnerability to (re)infection in long-followed PLWH and PWoH. The frequency of (re)infection by HCoVs in time can reveal if there is an increased chance for (re)infections in PLWH not on cART, compared to control PWoH. It is known that exposure to HCoVs occurs frequently, mostly in the winter season [17,18]. Importantly though, fluctuations in HCoV species dominance occurs [19], and comparison between groups therefore requires sampling in the same calendar years.

The Amsterdam Cohort Studies (ACS) is an ongoing open cohort study on the epidemiology of HIV-1 infection and other sexually and blood-transmitted infection (STI) among men who have sex with men (MSM) in Amsterdam [20]. The study started to enroll both PLWH and PWoH in 1984. In 1996 cART became generally available [21]. From a previous study we know that HCoV (re)infections occur regularly in the 1980s and 1990s [17]. By comparing the incidence of HCoV infection of PLWH not on cART to a comparable group of PWoH followed in the same calendar years, we can approximate the vulnerability of the first group for a reinfection by HCoVs as a proxy of PLWH without access to cART in modern times.

## 2. Methods

### 2.1. Study participants

The overall aim of the ACS is to investigate the epidemiology, psychosocial determinants, course of infection, and pathogenesis of HIV-1 infection, and of sexually, blood or otherwise transmitted infections other than HIV-1, and to evaluate the effects of interventions. MSM were eligible for inclusion when being over 18 years of age, taking part in sexual activities with other men six months prior to recruitment and residing in (or have been in contact with someone residing in) Amsterdam [21,22]. PLWH and PWoH were followed up at least every six months. During each visit, blood was collected for testing and storage, with serum stored at -80°C until use. The CD4+ T cell and CD8+ T cell counts were routinely measured for PLWH by flow cytometry [21]. Recruitment and follow up of ACS participants started in October 1984 and continues to the present day. At the first visit, socio-demographic, clinical and behavioral data were recorded for each person, including age, country of birth, current city of residence, latest education and income level, living arrangement (alone/with others), full-time activity (work, study, volunteering, etc.), smoking history, and the estimated number of sex partners in the past six months. Recruitment and sampling of the participants in our study started 02/11/1984 and the last sample in the current study was collected on 18/07/1994.

The required number of participants to be included in the current study was based on power calculation. A previous study on the interval length of any HCoV reinfection in PWoH (n = 10) revealed a median ± SD in interval between reinfections of 34.4 ± 20.6 months [17]. The median reinfection interval in untreated PLWH is hypothesized to be shorter due to immune deficiency and we predicted a 50% decrease mean interval. Using α of 0.05, power (β) of 80%, and the enrollment ratio of 1:1 for PLWH and PWoH, we calculated that we need 25 PLWH and 25 PWoH in the current study. Ideally the complete full follow up time from 1984 till the start of cART in 1996 was included in the study, however, in untreated PLWH this can be done only if we would select PLWH known as “long term non-progressors” or “elite controllers”, because a “typical progressor” dies before a 10 year follow up can be reached. To avoid bias towards the slow progressors or elite controllers we limited our study to only 7–8 years follow up, to be able to include typical progressors. The other PLWH and PWoH were subsequently also followed for not more than the 7 or 8 years, because it is important to measure groups in the same calendar years and the same follow up time. We managed to include 15 PLWH that either started or developed severe immune deficiency during follow up. For most of them the final sample was the last sample before death. Some PLWH (n = 8; numbers 3, 5, 6, 11, 14, 15, 24, 25) were at some moment on mono-antiretroviral medication using azidothymide. No effect of monotherapy is however expected as resistance to azidothymide develops within months on monotherapy. Combination therapy was not yet available during the study period, thus all PLWH were considered “not on cART”. PWoH had to have HIV-1 seronegative test results throughout the complete study duration. All serum samples (PLWH and PWoH) were processed and stored under the same conditions.

### 2.2. Ethics statement

In 1984 the study was approved by the Institutional Board of the GG&GD Amsterdam (29/10/1984, 011.8.07), followed by approval by the Medical Ethics Committee of the Amsterdam University Medical Center of the University of Amsterdam, the Netherlands (20/08/2007, MEC 07/182). Participants provided written informed consent and received no monetary incentive. Authors had no access to information that could identify individual participants during or after data collection.

### 2.3. Partial HCoV nucleocapsid multiplex assay

The antibody level against the nucleocapsid (N) protein of endemic HCoV was measured using an in-house multiplex assay. Anti-N antibody rise is a robust indication of infection by a HCoV, equally good as neutralization tests, as we previously demonstrated [17]. We specifically used the partial HCoV N antigen consisting of the C-terminal domain (NCt) for HCoV-NL63, HCoV-229E, and HCoV-OC43, as well as the linker-and-Ct-domain (NLCt) for HCoV-HKU1 as these antigens are sufficiently sensitive for their matched viruses while being less confounded by cross-reactivity within HCoV genus (HCoV-NL63 and HCoV-229E, both *Alphacoronavirus*, or HCoV-OC43 and HCoV-HKU1, both *Betacoronavirus*) [17,23].

The HCoV antigen NL63-NCt, 229E-NCt, OC43-NCt, and HKU1-NLCt were expressed as previously described [8,23]. The antigens were coupled to Luminex Magplex beads as previously described [23] at the ratio of 583 pmol antigens to 12.5 million beads. The multiplex assay was done with Magplex beads with no coupled antigen (no-antigen assay) included in each run as the negative control [23]. All serum samples from one participant were grouped within one assay plate and assayed in duplicate with the duplicates tested on a separate plate. The geometric mean was calculated from the duplicate values to obtain the final assay value per each time point and presented as median fluorescence intensity (MFI).

### 2.4. Data analysis

We defined antibody dynamics as assay signal fold-change values from two subsequent time points. We established the fold-change value ≥ 1.8 as a cut-off for HCoV (re)infection (S1 Data, S1 Table, S2 Table). A minimum cut-off of 10% difference between fold-change in one time point was implemented to account for possible within-genus cross-reactivity [17]. The infection date was defined as the midpoint between the study visit with a significant anti-HCoV IgG rise and the previous study visit included in this study.

HCoV infections in adults can be considered as reinfections, as it is known that the first HCoV seroconversion in a human lifetime occurs during childhood [8,9]. Reinfections in this study were specifically defined as an infection that was preceded by a previous infection by the same HCoV species, and interval length from previous to reinfection was defined as the interval in days between the estimated dates of two same-species infections. Thus, an infection without a preceding same-species infection is considered as “an infection”.

To evaluate the effect of immune cell depletion as the consequence of HIV-1 infection on PLWH, we made a sigmoid 4PL regression curve on elapsed days since first CD4+ T cell measurement (x values) versus all available CD4+ T cell count data (y values). A CD4+ T count of 350 cell/mm3 or less was found to be associated with a higher risk of AIDS and death [24]. Thus, in this study, a CD4+ T count of 350 cell/mm3 was established as the threshold of immune deficiency. The elapsed days between the first study visit and the timepoint when CD4+ T cell count = 350 cells/mm3 was interpolated from the curve including all CD4 counts of a person and used to determine the date when an individual reached immune deficiency. All data points of PLWH were pooled and then split into two groups (CD4+ T-cell count > 350 cell/mm3 (without immune deficiency) and ≤ 350 cell/mm3 (with immune deficiency)), and the HCoV infections were counted per groups.

We compared the median of all assay signal values per participants, as well as all antibody fold-change values ≥ 1.8, between PLWH and PWoH. In cases of HCoV infections with a known elapsed time from the preceding infection, the reinfection interval length in days was also compared between PLWH and PWoH. Furthermore, we compared the median of all-signal values per participants, as well as all antibody fold-change values ≥ 1.8, between PLWH with and without immune deficiency. This comparison was done as the serum IgG fold-change cut-off ≥ 1.8 - recognizing HCoV infection - was determined by testing immunocompetent people [17], and it could be that PLWH with low CD4+ T cell counts may not produce enough antibodies to mark infections by serum antibody-dynamics testing. All aforementioned comparisons (on continuous variables, median assay signal values per person, antibody fold-change values ≥ 1.8, and reinfection interval length) were done using Mann-Whitney U test.

Infections by any HCoV on either PLWH or PWoH, as well as infections in PLWH with CD4+ T cell count above and below the immune deficiency cut-off, were expressed as an incidence rate, calculated by dividing the number of infections with person time-at-risk of infection in years (1 year = 365.25 days), and the 95% confidence interval (CI) surrounding it was calculated using Poisson distribution. The time-at-risk for HCoV infection was defined as the whole follow-up period [17]. The HCoV infection incidence rate between PLWH and PWoH, as well as the incidence rate between PLWH with CD4+ T-cell counts of > 350 cell/mm3 and ≤ 350 cell/mm3, were compared using negative binomial regression to account for possible over-dispersion of values. The comparison of incidence rate was expressed as incidence rate ratio and 95% CI. Statistical analyses were done using SPSS Version 27 (IBM) and Prism version 9.5.1 (GraphPad).

## 3. Results

We included 25 PLWH and 25 PWoH who have participated in the ACS from the study conception (1984) for at least seven years. The socio-demographic and behavioral characteristics of PLWH and PWoH at the time of enrollment did not significantly differ (Table 1). We measured the level of serum anti-N IgG for all HCoVs (HCoV-NL63, HCoV-229E, HCoV-OC43, and HCoV-HKU1) of all available data points and observed significant (fold-change ≥ 1.8) IgG rises due to HCoV infection in both PLWH and PWoH (S1 Fig, S2 Fig). The no-antigen assay values (negative control) were stable and low throughout the years for all subjects, except for one participant (S1 Fig: Subject 21). For this participant, there was a background signal in the no-antigen assay and we excluded this subject from further analyses. The final study population therefore included 49 participants: 24 PLWH and 25 PWoH, with comparable cumulative time periods, 64,787 days (177 years) for PLWH and 69,106 days (189 years) for PWoH (Mann-Whitney U; p = 0.52). The PLWH were observed for a median duration of 2,967 days per participant (Interquartile range (IQR) = 2,579 – 2,757 days) and PWoH participants for a median duration of 2,762 days (IQR = 2,730 – 2,880 days) per participant (S3 Table, S4 Table). Included visits were spaced every six months for both study groups; median of days between sampling for PLWH and PWoH were 182 (IQR 175–189) days and 182 (IQR 177–189) days, respectively).

**Table 1:** Socio-demographic characteristics of participants by HIV-1 status.

Variables^a^	People without HIV-1 (PWoH; *n* = 25)	People living with HIV-1 (PLWH; *n* = 24)	p-value^a^
Age at enrollment, in years^b^	35 [32 –40 ]	35 [32 –38 ]	0.644
Born in the Netherlands^c^ Not born in the Netherlands	21 (91%) 2 (8%)	19 (95%) 1 (4%)	1
*Unknown*	*2 (8%)*	*4 (17%)*	*–*
“High” education completed^c,d^ “Low” or “intermediate” education completed	8 (32%) 11 (58%)	9 (39%) 13 (59%)	1
*Unknown*	*6 (24%)*	*2 (8%)*	*–*
Living alone^c^ Living with others	12 (63%) 7 (37%)	13 (57%) 10 (43%)	0.757
*Unknown*	*6 (24%)*	*1 (4%)*	–
Work/study full time^c^ Part-time/not working	14 (74%) 5 (26%)	20 (87%) 3 (13%)	0.433
*Unknown*	*6 (24%)*	*1 (4%)*	–
Smoking daily^c^ Non-smoking	12 (67%) 6 (33%)	13 (59%) 9 (41%)	0.747
*Unknown*	*7 (28%)*	*2 (8%)*	*–*
Number of sex partner(s) in the past 6 months^b^	10 [4 –15 ]	14 [8 –20 ]	0.097
Treatment with azidothymide monotherapy during follow up	N.A.	8 (33%)	–
Starting or reaching CD4 T-cell numbers below 350 cells/mm^3^	N.A.	15 (63%)	–

a Data is presented as median (IQR) for continuous variables or count (percentage) for categorical variables. P-values are determined by Mann-Whitney U test for continuous variables or Fisher’s exact test for categorical variables.

b Follow-up duration in days, age at enrollment in years, and sex partner(s) in past 6 months are presented as median [IQR].

c Information on these categorical variables was unavailable for some participants, and the percentage of missing data is given in the “Unknown” row directly below. For each categorical variables, percentages were calculated and statistical analyses were done from all available data.

d Higher education is defined according to the Dutch Standard Classification of Educational (SOI) [25], which is based on UNESCO International Standard Classification of Education Fields of Training and Education 2013 (ISCED-F 2013) [26]. Educational level was categorized as “low” (i.e., early childhood, primary or lower secondary education), “intermediate” (i.e., upper secondary or post-secondary education), and “high” (i.e., short cycle, bachelor, master or doctoral education).

**Fig 1:**
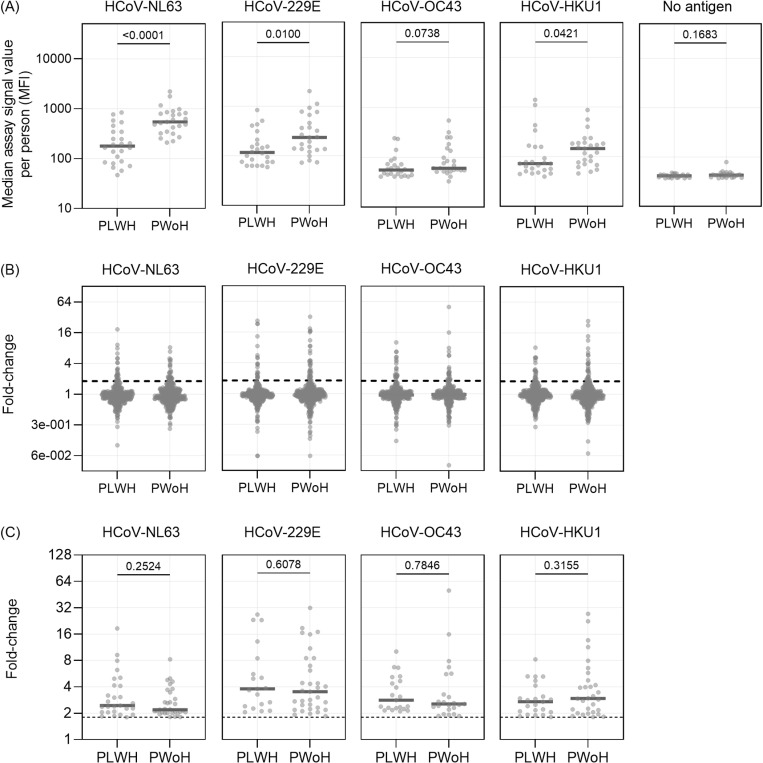
Antibody levels and dynamics from all data points compared between people living with HIV-1 (PLWH) and people without HIV-1 (PWoH). (A) The median of multiplex assay signal values from each participant was compared between PLWH and PWoH for each human coronavirus (HCoV) antigen and no-antigen assay using Mann-Whitney U test. Values are presented as median fluorescence intensity (MFI). Each dot represents a participant and the grey solid line represent the median value of all population. (B) & (C) The fold-change values of all time points (B) or time points when the values ≥ 1.8 (C) inr PLWH and PWoH for four HCoV antigen assays. Fold-change values were calculated as the assay signal value ratio from two subsequent time points. Dashed vertical line denotes the fold-change cut-off of 1.8 to represent infection. Each dot represents one observation, and the solid grey lines denote the median value of the population. The p-values for the comparison of HCoV infection fold-changes in PLWH and PWoH (C) were calculated using the Mann-Whitney U test.

We compared the median antibody level (multiplex assay signal values) from all time points per subject between PLWH (*n* participants = 24, *n* visits = 384) and PWoH (*n* participants = 25, *n* visit = 382). The median antibody level of PLWH as a group was significantly lower than the levels of PWoH across all antigens (HCoV-NL63 p < 0.001, HCoV-229E p = 0.01, HCoV-OC43 p = 0.07, HCoV-HKU1 p = 0.04; Fig 1A). Next, we calculated the antibody level fold-change value between two consecutive time points to determine the antibody dynamics. As expected, most fold-change values for both groups from all visits were concentrated around 1 (Fig 1B), but, importantly, the fold-change values that indicate HCoV infections (fold-change ≥ 1.8) were not significantly different between PLWH and PWoH on all HCoV antigens (HCoV-NL63 p = 0.25, HCoV-229E p = 0.60, HCoV-OC43 p = 0.78, HCoV-HKU1 p = 0.32; Fig 1C). This indicates that even in PLWH with lower antibody baseline level, an increase of HCoV N antibodies can be used as a marker for infection in both groups.

We found a total of 73 HCoV infections (*n* HCoV-NL63 = 22, *n* HCoV-229E = 17, *n* HCoV-OC43 = 18, and *n* HCoV-HKU1 = 16) in PLWH (S3 Table). The number of infections by HCoVs among PWoH was 84 (*n* HCoV-NL63 = 19, *n* HCoV-229E = 27, *n* HCoV-OC43 = 22, and *n* HCoV-HKU1 = 21) (S4 Table). The HCoV infection incidence rate was 0.41 (95% CI = 0.26 – 0.56) and 0.47 (95% CI = 0.35 – 0.63) infection per person-year for PLWH and PWoH, respectively. Negative binomial regression analysis revealed that the incidence rate ratio of HCoV infection in PLWH compared to PWoH was 0.83 (95% CI = 0.58-1.19, *p*-value = 0.311), suggesting that the incidence of endemic HCoVs infection were comparable between both groups.

**Fig 2:**
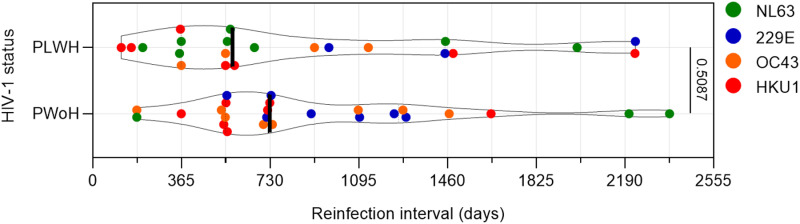
Reinfection interval length between PLWH and PWoH. Violin plot depicting the distribution of reinfection interval length between HIV-1 serostatus groups. Each dot represents one reinfection interval (same HCoV species) and colored differently to represent the HCoV species. Vertical line: median of each dataset. All p-values were calculated using the Mann-Whitney U test.

For 22 out of 73 infections among PLWH, the interval lengths between the current infection and the preceding same-species infection could be determined. The median interval length between infections for all PLWH was 574 days [IQR = 364 – 1,370 days]. These reinfections consisted of 8 HCoV-NL63 reinfections, 3 HCoV-229E reinfections, 4 HCoV-OC43 reinfections and 7 HCoV-HKU1 reinfections. For PWoH, 25 reinfections with determinable interval length were found, with median interval length of 728 days [IQR = 548 – 1,240 days]. These reinfections consisted of 4 HCoV-NL63 reinfections, 6 HCoV-229E reinfections, 8 HCoV-OC43 reinfections and 7 HCoV-HKU1 reinfections. Using these reinfection interval data, we found no significant difference in length of the reinfection interval across serostatus groups (Mann-Whitney U test p = 0.509) (Fig 2), suggesting that PLWH do not mount a less functional protection against HCoVs compared to PWoH.

Finally, we compared the incidence rate of HCoV infections among PLWH with and without immune deficiency (<or> CD4^+^ T cell count of 350 cell/mm^3^ respectively). Based on the interpolated CD4^+^ T cell count, two participants entered the study with immune deficiency, while 13 others crossed this CD4^+^ T cell cut-off during follow-up (S5 Table). The median duration in days from study entry to the time of the CD4^+^ T cell cut-off was 1311 days [IQR = 763–2028 days], and the median age of PLWH with (interpolated) CD4^+^ T-cell count at this threshold was 36 years [IQR = 34-39.5 years] among the 15 participants who were or became immunodeficient (Table 1). We found no significant difference in both the median assay signal values per participant and all fold change values from all infections (fold-change ≥ 1.8) between PLWH with CD4^+^ T cell count > 350 cell/mm^3^ and those with ≤ 350 cell/mm^3^ (S3A Fig, S3B Fig, and S3C Fig). We therefore conclude that reinfections can still be detected by means of measuring antibody dynamics when CD4 T-cell counts are low.

The total follow-up time of PLWH without and with immune deficiency is 44,292 days (121 years) and 20,495 days (56 years), respectively (S6 Table). Fifty-five infections were found among PLWH without immune deficiency, while 18 infections were counted with immune deficiency. This translates into the incidence rate of 0.46 (95% CI = 0.32 – 0.60) and 0.32 (95% CI = 0.17 – 0.47) HCoV infection per person-year without and with CD4^+^ T cell depletion, respectively (S6 Table). Negative binomial regression analysis showed that the incidence rate ratio of HCoV infection in PLWH with immune deficiency compared to without immune deficiency was 0.69 (95% CI = 0.38-1.25, *p*-value 0.215), suggesting that the incidence of endemic HCoV infection did not change significantly in PLWH with and without immune deficiency.

## 4. Discussion

In this study, the HCoV infection incidence rate did not differ between PLWH not on cART and PWoH. We also found, upon further investigation, that the incidence rate of endemic HCoV infections among PLWH with immunodeficiency (CD4^+^ T cells ≤ 350 cell/mm^3^) was not significantly different from without immunodeficiency. These findings suggest that adult PLWH seem not more vulnerable to a repeated HCoV infection, even when their CD4^+^ T cells are low due to untreated HIV-1 infection. As most recent SARS-CoV-2 studies enrolled PLWH already on cART with a relatively high CD4^+^ T cell counts [2,3,27], our study bridged the coronavirus knowledge gap by studying HCoV infections in PLWH with low CD4^+^ T cell counts.

Our results also show that PLWH were similarly protected from HCoV infection as PWoH despite having lower level of circulating serum IgG recognizing HCoVs. Apparently the baseline concentration of antibodies does not dictate vulnerability for reinfection. Since all four endemic HCoVs are encountered for the first time early in life [8,17], we suggest that the reactivation of immune memory upon (re)infection provides the “normal” short lived protection in people who later in life acquired a CD4 T-cell mediated immune deficiency.

We established that low CD4 + T-cell numbers do not seem to render a person more susceptible to reinfection by HCoVs. This finding is unexpected, but not unexplainable. In PLWH not on cART their immunity is mostly hampered due to the loss of CD4+ T-cells. B-cell numbers are less affected. This is illustrated by the frequently found AIDS defining illnesses *Pneumocystis carinii* pneumonia and esophageal candidiasis by *Candida albicans* [28], opportunistic fungal infections that require cell-mediated immunity as defense. Coronavirus clearance or protection from reinfection may depend less on T-cell immunity, more on humoral immunity, as was shown with the first mRNA based vaccines for SARS-CoV-2 [29].

In our controls (PWoH), protection from reinfections waned fast, with sometimes reinfection within one year (3 out of 25 reinfections (12%)). This number was not particularly different from our earlier finding (11 of 49 (22%) in PWoH [17]). It needs mentioning here that HCoVs are seasonal coronaviruses, with most circulation in the winter months [17]. If vulnerability to infection in PLWH would have waned faster, with for instance being susceptible to infection within 6 or 9 months, there is a chance that these more vulnerable PLWH may not have been exposed to HCoVs in these 6–9 months if this was in spring, summer or fall. In that case we see reinfections occurring after 12 months when it is again the winter season. We found a total of 7 out of 22 (32%), within-one-year reinfections in PLWH. Some of these may reflect the “season-effect” and the interval might have been shorter if HCoVs would have circulated the whole year round. Future studies may consider measuring serum HCoV-neutralizing capacity in PLWH in the first months after HCoV-infection and the pace of waning neutralization. In such a study, monthly sampling would be needed to allow quantification of the pace with which neutralizing antibodies wane, which may however be difficult in a prospective study because it is unethical to not provide cART to PLWH.

We have previously published on reinfections by HCoVs with a follow up time between 10–16 years in PWoH. The reinfections in that study occur with a median ± SD intervals of 34.4 ± 20.6 months [17]. In another study we measured 17 years of follow up in PWoH and we found a median time till reinfection of 43 months [IQR 23 months – 68 months] [30]. In the current study the median reinfection interval was 24 months [IQR 18 months – 40 months] for PWoH, which may seem somewhat lower than the numbers we previously published. This was however expected since an interval between reinfections cannot exceed the follow up time, and therefore intervals > 7 years could not have been found in our current study. The dependence of the median interval on follow up time requires caution, and it makes comparison of studies with varying durations of follow up difficult to do. Most important in the present study is the comparison between PLWH and PWoH followed for the same number of years. The values we found, a median of 19 months [IQR 12 months – 45 months] for PLWH versus 24 months [IQR 18 months – 40 months] for PWoH, were not significantly different between the two groups.

As SARS-CoV-2 has now been considered endemic in worldwide, future reinfections might also occur equally in PLWH and PWoH. Still, untreated PLWH who encountered SARS-CoV-2 for the first time when their CD4^+^ T cells were depleted might be unable to generate adequate antibody-mediated immunity against this virus. CD4^+^ T cells aid in co-stimulation of B cells to produce high affinity antibodies, especially IgGs [31]. Studies have indeed shown that PLWH on cART have lower memory B cell numbers and lower high-affinity IgG titer after SARS-CoV-2 infection or vaccination [32,33]. Considering that CD4^+^ T cell replenishment in response to cART treatment might be the cause of similar SARS-CoV-2 infection prevalence (and clinical outcomes) among PLWH and PWoH in most studies [2,3], and that the majority of PLWH with high CD4^+^ T cells respond well to SARS-CoV-2 vaccination including the third booster [27], a more thorough coverage of cART on PLWH worldwide will aid in further ensuring the immune-memory generation (and subsequent protection) against future SARS-CoV-2 reinfections in PLWH.

Our study benefits from enrolling both PLWH and PWoH groups from the same key population, namely MSM [34]. PLWH and PWoH in our study had comparable sociodemographic characteristics at enrollment such as age, education background, occupation, and living arrangement, and thus both groups can be considered comparable for study purposes.

There are some questions that our study could not answer. The first one is the effect of HIV-1 status and/or CD4+ T cell depletion on the disease caused by an HCoV infection. Symptoms of influenza-like-illness (fever, cough, myalgia, etc.) were documented for PWoH in ACS as these symptoms might indicate a primary HIV-1 infection [22], and they could be used to approximate the disease manifestation upon HCoV infection [30]. Unfortunately, no such record was available for the PLWH, as they entered the cohort already living with HIV-1. This is an important question to address in future studies, as PLWH contracting COVID-19 had a higher risk of hospitalization than PWoH [2]. Next to that, our study population comprises exclusively adult men. Further studies are needed to investigate whether sex would have an influence on vulnerability for HCoV infection among PLWH and also among PWoH.

## 5. Conclusion

We report in this study that the incidence rate of endemic HCoV infection in PLWH not on cART is not significantly different from that in PWoH. Furthermore, we found among PLWH not on cART that immune deficiency characterized by low CD4^+^ T-cell count did not seem to influence the incidence of endemic HCoV infection.

## Supporting information

S1 Data Determination of the antibody rise cut-off value to establish HCoV infection. (DOCX)

S2 Data Raw data of the study. (XLSX)

S1 Table Cross-tabulation of infections found with ELISA and multiplex assay across antigens and cut-off values. (DOCX)

S2 Table Sensitivity and specificity of the multiplex assay on different cut-off values. (DOCX)

S3 Table Follow-up duration and infection frequency by each and all HCoV on PLWH. (DOCX)

S4 Table Follow-up duration and infection frequency by each and all HCoV on PWoH. (DOCX)

S5 Table Measured and interpolated CD4 + T cell counts of PLWH. (DOCX)

S6 Table Follow-up time and infections identified in participants with HIV-1 at CD4 + cell count > 350 cell/mm^3^ and the cell count ≤ 350 cell/mm^3^. (DOCX)

S1 Fig Antibody dynamics and immune cell marker during the follow-up period for PLWH (subject 1–25). (DOCX)

S2 Fig Antibody dynamics for PWoH (subject 26–50). (DOCX)

S3 Fig The distribution of anti-HCoV specific antibody level of PLWH with CD4 + T cell count above and below 350 cell/mm^3^. (DOCX)
