# Supplementary material for: Untreated HIV-1 infection and low CD4+ T cell counts and their effect on endemic human coronavirus (re)infection
Source: PLOS Glob Public Health. 2025 Jun 18;5(6):e0004610. doi: 10.1371/journal.pgph.0004610 (PMC12176178; doi:10.1371/journal.pgph.0004610)
Supplement: S1 Data — (DOCX) [file pgph.0004610.s001.docx]

**Supplementary Material**

**Untreated HIV-1 infection and low CD4^+^ T cell counts and their effect on endemic HCoV (re)-infection**

Ferdyansyah Sechan, Anne W. M. van den Hurk, T. Sonia Boender, Maria Prins, Amy Matser, Margreet Bakker, Neeltje A. Kootstra, and Lia van der Hoek

**S1 Data**: **Determination of the antibody rise cut-off value to establish HCoV infection**

We previously established an ELISA protocol to detect the antibody rise as an indication of human coronavirus (HCoV) infection. Antibody dynamics was defined as the assay fold-change values from two subsequent time points, with a fold-change cut-off of 1.40 deemed as an infection [1]. We used the C-terminal (Ct) domain for the nuclecapsid (N) protein (NL63-NCt, 229E-NCt, and OC43-NCt antigen) for the HCoVs except HCoV-HKU1, in which the linker (L) domain of the N protein was also included (HKU1-NLCt) [2,3]. When tested on serum samples of people with confirmed infection against endemic HCoVs, the sensitivity of 91% for NL63-NCt, 81% for 229E-NCt, 71% for OC43-NCt, and 29% for HKU-NLCt was found [1,2]. For this study, these four antigens were implemented in the multiplex-based assay (Luminex). Due to the assay being more sensitive than ELISA, a new cut-off for antibody rise in response to infection was established.

A total of 380 serum samples (time points) from 10 HIV-1 seronegative men from the Amsterdam Cohort Studies (ACS) were included in this study. These serum samples were previously tested with ELISA and both the ELISA signals and the fold-change values were known [1]. These samples were then re-assayed with the partial N multiplex assay, and three cut-off values were evaluated: 1.4, 1.8, and 2.2. The infection by each HCoV in a given time point was compared between ELISA and multiplex output, with the infections from ELISA data assigned as the actual infections. The sensitivity (the ratio between true positive and total infection from ELISA) and specificity (the ratio between true negative and total non-infection from ELISA) of the multiplex assay compared to ELISA were then evaluated.

The number of infection (fold-change at least or above cut-off) was generally higher in the multiplex assay with fold-change value cut-off of 1.4, and raising the cut-off to 1.8 and 2.2 steadily decreased the infection number by multiplex that was not detected by ELISA (**S1 Table**). The sensitivity averaged across the four antigens was 0.68 for cut-off 1.4, 0.60 for cut-off 1.8, and 0.55 for cut-off 2.2 (**S2 Table**). The NL63-NCt antigen generally had the highest sensitivity while HKU1-NLCt had the lowest sensitivity. The specificity averaged across the four antigen was 0.86 for cut-off 1.4, 0.93 for cut-off 1.8, and 0.95 for cut-off 2.2, with the specificity value of each antigen-cut-off combinations consistently above 0.80 (**S2 Table**). Based on this evaluation, the new cut-off of 1.8 was established for the multiplex assay, as it offers a balance between sensitivity and specificity.

**References:**

1. Edridge AWD, Kaczorowska J, Hoste ACR, Bakker M, Klein M, Loens K, et al. Seasonal coronavirus protective immunity is short-lasting. Nat Med. 2020;26: 1691–1693. doi:10.1038/s41591-020-1083-1

2. Sechan F, Grobben M, Edridge AWD, Jebbink MF, Loens K, Ieven M, et al. Atypical antibody dynamics during human coronavirus HKU1 infections. Front Microbiol. 2022;13: 1–9. doi:10.3389/fmicb.2022.853410

3. Dijkman R, Jebbink MF, Gaunt E, Rossen JWA, Templeton KE, Kuijpers TW, et al. The dominance of human coronavirus OC43 and NL63 infections in infants. Journal of Clinical Virology. 2012;53: 135–139. doi:10.1016/j.jcv.2011.11.011
