# Supplementary material for: Untreated HIV-1 infection and low CD4+ T cell counts and their effect on endemic human coronavirus (re)infection
Source: PLOS Glob Public Health. 2025 Jun 18;5(6):e0004610. doi: 10.1371/journal.pgph.0004610 (PMC12176178; doi:10.1371/journal.pgph.0004610)
Supplement: S1 Table — (DOCX) [file pgph.0004610.s003.docx]

**Supplementary Material**

**Untreated HIV-1 infection and low CD4^+^ T cell counts and their effect on endemic HCoV (re)-infection**

Ferdyansyah Sechan, Anne W. M. van den Hurk, T. Sonia Boender, Maria Prins, Amy Matser, Margreet Bakker, Neeltje A. Kootstra, and Lia van der Hoek

**S1 Table. Cross-tabulation of infections found with ELISA and multiplex assay across antigens and cut-off values.**

|  | | **Multiplex cut-off 1.4** | | **Multiplex cut-off 1.8** | | **Multiplex cut-off 2.2** | |
| --- | --- | --- | --- | --- | --- | --- | --- |
| ***NL63-NCt*** | | **0** | **1** | **0** | **1** | **0** | **1** |
| **ELISA cut-off 1.4*** | **0** | 295 | 57 | 319 | 33 | 330 | 22 |
|  | **1** | 4 | 24 | 6 | 22 | 7 | 21 |
| ***229E-NCt*** | | **0** | **1** | **0** | **1** | **0** | **1** |
| **ELISA cut-off 1.4*** | **0** | 300 | 40 | 317 | 23 | 324 | 16 |
|  | **1** | 11 | 29 | 15 | 25 | 17 | 23 |
| ***OC43-NCt*** | | **0** | **1** | **0** | **1** | **0** | **1** |
| **ELISA cut-off 1.4*** | **0** | 317 | 35 | 338 | 14 | 346 | 6 |
|  | **1** | 11 | 17 | 14 | 15 | 15 | 13 |
| ***HKU1-NLCt*** | | **0** | **1** | **0** | **1** | **0** | **1** |
| **ELISA cut-off 1.4*** | **0** | 297 | 66 | 332 | 31 | 342 | 21 |
|  | **1** | 8 | 9 | 9 | 8 | 10 | 7 |
| *Infection from ELISA is assigned as the actual infection. | | | | | | | |
