# Supplementary material for: Untreated HIV-1 infection and low CD4+ T cell counts and their effect on endemic human coronavirus (re)infection
Source: PLOS Glob Public Health. 2025 Jun 18;5(6):e0004610. doi: 10.1371/journal.pgph.0004610 (PMC12176178; doi:10.1371/journal.pgph.0004610)
Supplement: S2 Table — (DOCX) [file pgph.0004610.s004.docx]

**Supplementary Material**

**Untreated HIV-1 infection and low CD4^+^ T cell counts and their effect on endemic HCoV (re)-infection**

Ferdyansyah Sechan, Anne W. M. van den Hurk, T. Sonia Boender, Maria Prins, Amy Matser, Margreet Bakker, Neeltje A. Kootstra, and Lia van der Hoek

**S2 Table. Sensitivity and specificity of the multiplex assay on different cut-off values.**

|  | **Multiplex cut-off 1.4** | | **Multiplex cut-off-1.8** | | **Multiplex cut-off 2.2** | |
| --- | --- | --- | --- | --- | --- | --- |
|  | **Sensitivity** | **Specificity** | **Sensitivity** | **Specificity** | **Sensitivity** | **Specificity** |
| **NL63-NCt** | 0.86 | 0.84 | 0.79 | 0.91 | 0.75 | 0.94 |
| **229E-NCt** | 0.73 | 0.88 | 0.63 | 0.93 | 0.58 | 0.95 |
| **OC43-NCt** | 0.61 | 0.90 | 0.52 | 0.96 | 0.46 | 0.98 |
| **HKU1-NLCt** | 0.53 | 0.82 | 0.47 | 0.91 | 0.41 | 0.94 |
| **Average** | 0.68 | 0.86 | 0.60 | 0.93 | 0.55 | 0.95 |
