# Supplementary material for: Untreated HIV-1 infection and low CD4+ T cell counts and their effect on endemic human coronavirus (re)infection
Source: PLOS Glob Public Health. 2025 Jun 18;5(6):e0004610. doi: 10.1371/journal.pgph.0004610 (PMC12176178; doi:10.1371/journal.pgph.0004610)
Supplement: S3 Table — (DOCX) [file pgph.0004610.s005.docx]

**Supplementary Material**

**Untreated HIV-1 infection and low CD4^+^ T cell counts and their effect on endemic HCoV (re)-infection**

Ferdyansyah Sechan, Anne W. M. van den Hurk, T. Sonia Boender, Maria Prins, Amy Matser, Margreet Bakker, Neeltje A. Kootstra, and Lia van der Hoek

**S3 Table. Follow-up duration and infection frequency by each and all HCoV on PLWH.**

| **People with HIV-1** | **HIV** | **Start month-year** | **End month-year** | **Follow-up days** | **HCoV-NL63** | **HCoV-229E** | **HCoV-OC43** | **HCoV-HKU1** | **Total** |
| --- | --- | --- | --- | --- | --- | --- | --- | --- | --- |
| 01 | + | 11-1984 | 12-1991 | 2580 | 2 | 1 | 1 | 1 | 5 |
| 02 | + | 05-1985 | 07-1992 | 2609 | 0 | 0 | 2 | 0 | 2 |
| 03 | + | 01-1985 | 07-1992 | 2718 | 1 | 0 | 2 | 0 | 3 |
| 04 | + | 05-1985 | 10-1992 | 2681 | 0 | 0 | 0 | 0 | 0 |
| 05 | + | 11-1984 | 01-1992 | 2603 | 0 | 0 | 0 | 0 | 0 |
| 06 | + | 12-1984 | 12-1991 | 2556 | 3 | 1 | 1 | 3 | 8 |
| 07 | + | 06-1985 | 08-1992 | 2618 | 1 | 1 | 0 | 0 | 2 |
| 08 | + | 05-1985 | 11-1992 | 2741 | 0 | 0 | 0 | 0 | 0 |
| 09 | + | 12-1984 | 11-1991 | 2533 | 2 | 0 | 0 | 0 | 2 |
| 10 | + | 11-1984 | 11-1991 | 2568 | 1 | 1 | 3 | 1 | 6 |
| 11 | + | 01-1985 | 01-1993 | 2924 | 2 | 0 | 0 | 0 | 2 |
| 12 | + | 11-1984 | 11-1991 | 2565 | 0 | 2 | 1 | 3 | 6 |
| 13 | + | 11-1984 | 11-1991 | 2562 | 1 | 0 | 1 | 1 | 3 |
| 14 | + | 01-1985 | 08-1992 | 2791 | 0 | 2 | 0 | 1 | 3 |
| 15 | + | 11-1984 | 05-1992 | 2745 | 1 | 1 | 0 | 1 | 3 |
| 16 | + | 04-1985 | 08-1992 | 2680 | 0 | 1 | 0 | 0 | 1 |
| 17 | + | 01-1985 | 07-1992 | 2725 | 1 | 1 | 1 | 1 | 4 |
| 18 | + | 02-1985 | 08-1992 | 2735 | 0 | 1 | 1 | 0 | 2 |
| 19 | + | 12-1985 | 12-1993 | 2912 | 0 | 0 | 1 | 0 | 1 |
| 20 | + | 11-1984 | 12-1991 | 2574 | 0 | 1 | 0 | 0 | 1 |
| 21 | + | 01-1985 | 11-1992 | 2855 | NA | NA | NA | NA | NA |
| 22 | + | 02-1985 | 08-1992 | 2744 | 1 | 1 | 1 | 1 | 4 |
| 23 | + | 11-1984 | 10-1992 | 2898 | 2 | 1 | 1 | 0 | 4 |
| 24 | + | 11-1984 | 09-1992 | 2886 | 1 | 0 | 1 | 0 | 2 |
| 25 | + | 12-1984 | 09-1992 | 2839 | 3 | 2 | 1 | 3 | 9 |
| *Total (all subjects)* | | | | 67642 | NA | NA | NA | NA | NA |
| *Total (without subject 21)* | | | | 64787 | 22 | 17 | 18 | 16 | 73 |
| NA: Not applicable (the infection frequency of subject 21 could not be counted). | | | | | | | | | |
