# Supplementary material for: Untreated HIV-1 infection and low CD4+ T cell counts and their effect on endemic human coronavirus (re)infection
Source: PLOS Glob Public Health. 2025 Jun 18;5(6):e0004610. doi: 10.1371/journal.pgph.0004610 (PMC12176178; doi:10.1371/journal.pgph.0004610)
Supplement: S4 Table — (DOCX) [file pgph.0004610.s006.docx]

**Supplementary Material**

**Untreated HIV-1 infection and low CD4^+^ T cell counts and their effect on endemic HCoV (re)-infection**

Ferdyansyah Sechan, Anne W. M. van den Hurk, T. Sonia Boender, Maria Prins, Amy Matser, Margreet Bakker, Neeltje A. Kootstra, and Lia van der Hoek

**S4 Table. Follow-up duration and infection frequency by each and all HCoV on PWoH.**

| **People without HIV-1** | **HIV** | **Start month-year** | **End month-year** | **Follow-up days** | **HCoV-NL63** | **HCoV-229E** | **HCoV-OC43** | **HCoV-HKU1** | **Total** |
| --- | --- | --- | --- | --- | --- | --- | --- | --- | --- |
| 26 | - | 03-1985 | 04-1992 | 2572 | 0 | 2 | 0 | 0 | 2 |
| 27 | - | 03-1985 | 03-1993 | 2905 | 1 | 2 | 0 | 2 | 5 |
| 28 | - | 05-1985 | 11-1992 | 2762 | 1 | 2 | 2 | 0 | 5 |
| 29 | - | 05-1985 | 04-1993 | 2873 | 0 | 1 | 1 | 0 | 2 |
| 30 | - | 12-1984 | 12-1991 | 2557 | 1 | 0 | 1 | 1 | 3 |
| 31 | - | 01-1985 | 12-1992 | 2870 | 2 | 0 | 2 | 0 | 4 |
| 32 | - | 01-1985 | 01-1992 | 2565 | 1 | 1 | 0 | 1 | 3 |
| 33 | - | 09-1985 | 08-1993 | 2885 | 1 | 1 | 1 | 0 | 3 |
| 34 | - | 12-1984 | 12-1992 | 2912 | 2 | 1 | 1 | 1 | 5 |
| 35 | - | 12-1984 | 12-1992 | 2921 | 0 | 1 | 0 | 0 | 1 |
| 36 | - | 01-1985 | 12-1992 | 2880 | 1 | 1 | 0 | 3 | 5 |
| 37 | - | 03-1985 | 09-1992 | 2747 | 0 | 1 | 1 | 0 | 2 |
| 38 | - | 01-1985 | 07-1992 | 2734 | 1 | 2 | 0 | 3 | 6 |
| 39 | - | 12-1984 | 07-1992 | 2774 | 0 | 1 | 1 | 1 | 3 |
| 40 | - | 02-1985 | 02-1993 | 2920 | 0 | 2 | 0 | 2 | 4 |
| 41 | - | 11-1984 | 11-1992 | 2917 | 0 | 0 | 1 | 0 | 1 |
| 42 | - | 11-1985 | 06-1993 | 2762 | 2 | 1 | 1 | 1 | 5 |
| 43 | - | 05-1985 | 12-1992 | 2743 | 1 | 1 | 0 | 1 | 3 |
| 44 | - | 05-1985 | 11-1992 | 2749 | 0 | 2 | 0 | 1 | 3 |
| 45 | - | 05-1985 | 10-1992 | 2730 | 2 | 1 | 3 | 1 | 7 |
| 46 | - | 02-1985 | 08-1992 | 2731 | 0 | 0 | 0 | 1 | 1 |
| 47 | - | 06-1985 | 12-1991 | 2364 | 1 | 1 | 1 | 2 | 5 |
| 48 | - | 12-1986 | 07-1994 | 2779 | 0 | 1 | 0 | 0 | 1 |
| 49 | - | 05-1985 | 11-1992 | 2730 | 1 | 1 | 2 | 0 | 4 |
| 50 | - | 05-1985 | 11-1992 | 2724 | 1 | 1 | 4 | 0 | 6 |
| *Total* | | | | 69106 | 19 | 27 | 22 | 21 | 89 |
