# Supplementary material for: Untreated HIV-1 infection and low CD4+ T cell counts and their effect on endemic human coronavirus (re)infection
Source: PLOS Glob Public Health. 2025 Jun 18;5(6):e0004610. doi: 10.1371/journal.pgph.0004610 (PMC12176178; doi:10.1371/journal.pgph.0004610)
Supplement: S5 Table — (DOCX) [file pgph.0004610.s007.docx]

**Supplementary Material**

**Untreated HIV-1 infection and low CD4^+^ T cell counts and their effect on endemic HCoV (re)-infection**

Ferdyansyah Sechan, Anne W. M. van den Hurk, T. Sonia Boender, Maria Prins, Amy Matser, Margreet Bakker, Neeltje A. Kootstra, and Lia van der Hoek

**Table S5. Measured and interpolated CD4^+^ T cell counts of PLWH.**

| **People with HIV-1** | **Test frequency** | **CD4^+^ measurement** | | **Measured CD4^+^ count (cell/mm^3^)** | | | **Interpolated* CD4^+^ count (cell/mm^3^)** | | |
| --- | --- | --- | --- | --- | --- | --- | --- | --- | --- |
|  |  | **Start month-year** | **End month-year** | **Start** | **End** | **Median (IQR)** | **Start** | **End** | **Date CD4 ≤ 350 cell/mm^3^** |
| 1 | 29 | 06-1985 | 12-1991 | 810 | 620 | 800 (660-1080) | 1170 | 590 | NA |
| 2 | 41 | 02-1985 | 05-1992 | 480 | 20 | 80 (40-150) | 570 | 50 | 24-02-1986 |
| 3 | 45 | 05-1985 | 06-1992 | 790 | 10 | 210 (90-400) | 530 | 30 | 06-04-1989 |
| 4 | 40 | 05-1985 | 06-1993 | 1180 | 0 | 235 (48-400) | 970 | 40 | 23-11-1987 |
| 5 | 40 | 06-1985 | 12-1991 | 280 | 260 | 340 (288-473) | 350 | 350 | NA |
| 6 | 35 | 06-1985 | 11-1991 | 220 | 470 | 440 (350-560) | 730 | 450 | NA |
| 7 | 31 | 04-1985 | 08-1992 | 560 | 50 | 230 (145-330) | 690 | 90 | 04-03-1987 |
| 8 | 30 | 08-1985 | 11-1992 | 770 | 20 | 115 (43-260) | 330 | 30 | 07-08-1985 |
| 9 | 32 | 07-1985 | 10-1991 | 610 | 250 | 310 (170-465) | 710 | 130 | 27-05-1988 |
| 10 | 27 | 08-1985 | 11-1991 | 540 | 460 | 480 (390-620) | 870 | 350 | NA |
| 11 | 33 | 07-1985 | 01-1993 | 550 | 380 | 740 (550-910) | 930 | 510 | NA |
| 12 | 29 | 08-1985 | 09-1991 | 760 | 110 | 300 (200-570) | 640 | 150 | 05-06-1988 |
| 13 | 30 | 05-1985 | 10-1991 | 570 | 240 | 435 (340-578) | 660 | 340 | 21-07-1990 |
| 14 | 30 | 07-1985 | 08-1992 | 920 | 640 | 805 (675-918) | 980 | 710 | NA |
| 15 | 43 | 02-1985 | 04-1992 | 500 | 40 | 250 (195-345) | 370 | 70 | 01-04-1988 |
| 16 | 35 | 01-1985 | 08-1992 | 1190 | 430 | 630 (510-805) | 902 | 530 | NA |
| 17 | 31 | 07-1985 | 07-1992 | 630 | 100 | 400 (320-555) | 570 | 220 | 30-06-1990 |
| 18 | 32 | 05-1985 | 08-1992 | 810 | 310 | 605 (475-870) | 910 | 420 | NA |
| 19 | 34 | 12-1985 | 12-1993 | 490 | 100 | 650 (460-808) | 1090 | 180 | 09-11-1992 |
| 20 | 28 | 05-1985 | 12-1991 | 950 | 400 | 615 (395-840) | 1340 | 460 | NA |
| *21*** | *34* | *04-1985* | *11-1992* | *690* | *10* | *135 (30-530)* | *600* | *10* | *15-09-1988* |
| 22 | 46 | 08-1985 | 07-1992 | 440 | 370 | 300 (263-360) | 270 | 310 | 14-08-1985 |
| 23 | 33 | 08-1985 | 10-1992 | 630 | 250 | 470 (330-630) | 850 | 230 | 08-11-1990 |
| 24 | 34 | 05-1985 | 09-1992 | 1280 | 80 | 500 (120-1028) | 1140 | 90 | 10-06-1989 |
| 25 | 45 | 06-1985 | 09-1992 | 480 | 250 | 540 (430-650) | 750 | 330 | 07-05-1992 |
| * For each person with HIV-1, a regression line was made from elapsed time (x axis) and all available CD4^+^ T cell counts (y axis) using sigmoid 4PL approach. Date when CD4^+^ T cell count reached 350 cell/mm^3^ was interpolated from this line. NA is given when the regression line did not reach 350 cell/mm^3^. ** Excluded from further analysis due to background signal on the no-antigen assay. | | | | | | | | | |
