# Supplementary material for: Untreated HIV-1 infection and low CD4+ T cell counts and their effect on endemic human coronavirus (re)infection
Source: PLOS Glob Public Health. 2025 Jun 18;5(6):e0004610. doi: 10.1371/journal.pgph.0004610 (PMC12176178; doi:10.1371/journal.pgph.0004610)
Supplement: S6 Table — (DOCX) [file pgph.0004610.s008.docx]

**Supplementary Material**

**Untreated HIV-1 infection and low CD4^+^ T cell counts and their effect on endemic HCoV (re)-infection**

Ferdyansyah Sechan, Anne W. M. van den Hurk, T. Sonia Boender, Maria Prins, Amy Matser, Margreet Bakker, Neeltje A. Kootstra, and Lia van der Hoek

**Table S6. Follow-up time and infections identified in participants with HIV-1 at CD4^+^ cell count > 350 cell/mm^3^ and the cell count ≤ 350 cell/mm^3^.**

| **People with HIV-1** | **CD4 ≤ 350 cells/mm^3^** | **Follow-up time (in days)** | | | **Age (in years)** | | **Total human coronavirus infection** | | |
| --- | --- | --- | --- | --- | --- | --- | --- | --- | --- |
|  |  | **All** | **CD4 > 350** **cells/mm^3^** | **CD4 ≤ 350** **cells/mm^3^** | **At baseline** | **At CD4 = 350 cell/ mm^3^** | **All** | **CD4 > 350 cells/ mm^3^** | **CD4 ≤ 350 cells/ mm^3^** |
| 01 | No | 2,580 | 2,580 | NA | 36 | NA | 5 | 5 | NA |
| 02 | Yes | 2,609 | 290 | 2,319 | 35 | 35 | 2 | 1 | 2 |
| 03 | Yes | 2,718 | 1,527 | 1,191 | 36 | 40 | 3 | 2 | 1 |
| 04 | Yes | 2,681 | 907 | 1,774 | 34 | 36 | 0 | 0 | 0 |
| 05 | No | 2,603 | 2,603 | NA | 35 | NA | 0 | 0 | NA |
| 06 | No | 2,556 | 2,556 | NA | 37 | NA | 8 | 8 | NA |
| 07 | Yes | 2,618 | 618 | 2,000 | 33 | 34 | 2 | 0 | 2 |
| 08 | Yes* | 2,741 | NA | 2,741 | 50 | 50 | 0 | NA | 0 |
| 09 | Yes | 2,533 | 1,256 | 1,277 | 47 | 50 | 2 | 1 | 2 |
| 10 | No | 2,568 | 2,568 | NA | 40 | NA | 6 | 6 | NA |
| 11 | No | 2,924 | 2,924 | NA | 36 | NA | 2 | 2 | NA |
| 12 | Yes | 2,565 | 1,311 | 1,254 | 35 | 38 | 6 | 2 | 4 |
| 13 | Yes | 2,562 | 2,070 | 492 | 36 | 41 | 3 | 3 | 0 |
| 14 | No | 2,791 | 2,791 | NA | 40 | NA | 3 | 3 | NA |
| 15 | Yes | 2,745 | 1,229 | 1,516 | 30 | 33 | 3 | 2 | 1 |
| 16 | No | 2,680 | 2,680 | NA | 45 | NA | 1 | 1 | NA |
| 17 | Yes | 2,725 | 1,985 | 740 | 33 | 38 | 4 | 3 | 0 |
| 18 | No | 2,735 | 2,735 | NA | 35 | NA | 2 | 2 | NA |
| 19 | Yes | 2,912 | 2,524 | 388 | 30 | 36 | 1 | 1 | 0 |
| 20 | No | 2,574 | 2,574 | NA | 28 | NA | 1 | 1 | NA |
| 22 | Yes* | 2,744 | NA | 2,744 | 27 | 27 | 4 | NA | 0 |
| 23 | Yes | 2,898 | 2,184 | 714 | 49 | 54 | 4 | 3 | 0 |
| 24 | Yes | 2,886 | 1,678 | 1,208 | 30 | 34 | 2 | 1 | 1 |
| 25 | Yes | 2,839 | 2,702 | 137 | 32 | 39 | 9 | 8 | 0 |
| ***Total*** | | 64,787 | 44,292 | 20,495 | NA | NA | 73 | 55 | 18 |
| NA: Not applicable (the participant did not have the CD4^+^ T-cell count either above or below 350 cell/mm^3^ during follow-up). | | | | | | | | | |
| * Participants entered the ACS cohort already with the interpolated CD4^+^ T-cell count of ≤ 350 cell/mm^3^. | | | | | | | | | |
