# Supplementary material for: Untreated HIV-1 infection and low CD4+ T cell counts and their effect on endemic human coronavirus (re)infection
Source: PLOS Glob Public Health. 2025 Jun 18;5(6):e0004610. doi: 10.1371/journal.pgph.0004610 (PMC12176178; doi:10.1371/journal.pgph.0004610)

**Supplementary Material**

**Untreated HIV-1 infection and low CD4^+^ T cell counts and their effect on endemic HCoV (re)-infection**

Ferdyansyah Sechan, Anne W. M. van den Hurk, T. Sonia Boender, Maria Prins, Amy Matser, Margreet Bakker, Neeltje A. Kootstra, and Lia van der Hoek


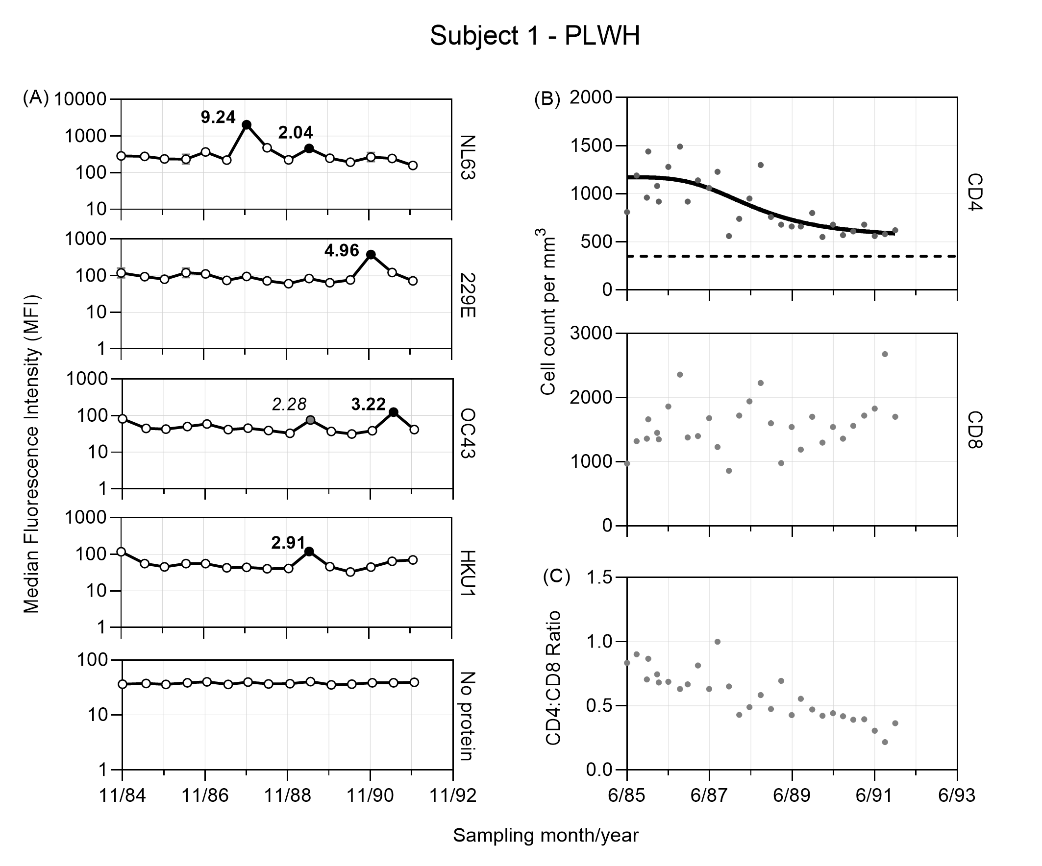


**S1 Fig. Antibody dynamics and immune cell marker during the follow-up period for PLWH (subject 1-25, see next pages for subjects 2-25).**

(A) Antibody dynamics of partial nucleocapsid antigen of endemic HCoV throughout time. Data is presented as geometric mean ± SD of two technical replicates for each serum sample in median fluorescence intensity (MFI). White dots: fold-change below 1.8. Black dots: fold change ≥ 1.80 as the indication of infection with fold change values given in black and bold above the data points. Gray dots: fold change ≥ 1.80 but not counted as infection due to either being cross-reaction (within-genus fold-change difference > 10%) or no difference could be made (within-genus fold-change difference: ≤ 10%); fold-change values are given in black and italic above the data points. Vertical dashed line (if present) represents the date when interpolated CD4^+^ T cell count = 350 cell/mm^3^.

(B) CD4^+^ and CD8^+^ T cell counts per subject throughout follow-up. Each dot represents measured values in cell/mm^3^. Solid curve denotes the sigmoid 4PL regression curve for CD4^+^ T cell count. Horizontal dashed line (if present) represents cell count = 350 cell/mm^3^. points. Vertical dashed line (if present) represents the date when interpolated CD4^+^ T cell count = 350 cell/mm^3^. (C) CD4^+^/CD8^+^ cell ratio per subject. Each dot represents measured values.

**S1 Fig (continued).**

**
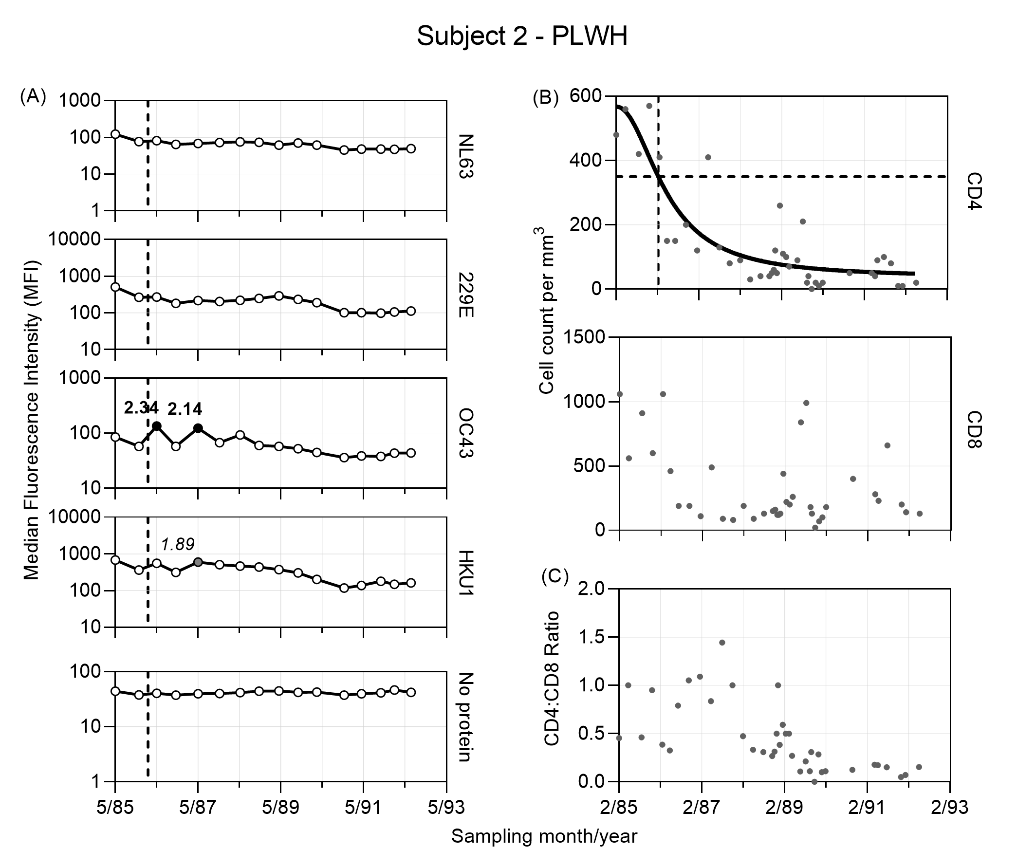
**

**
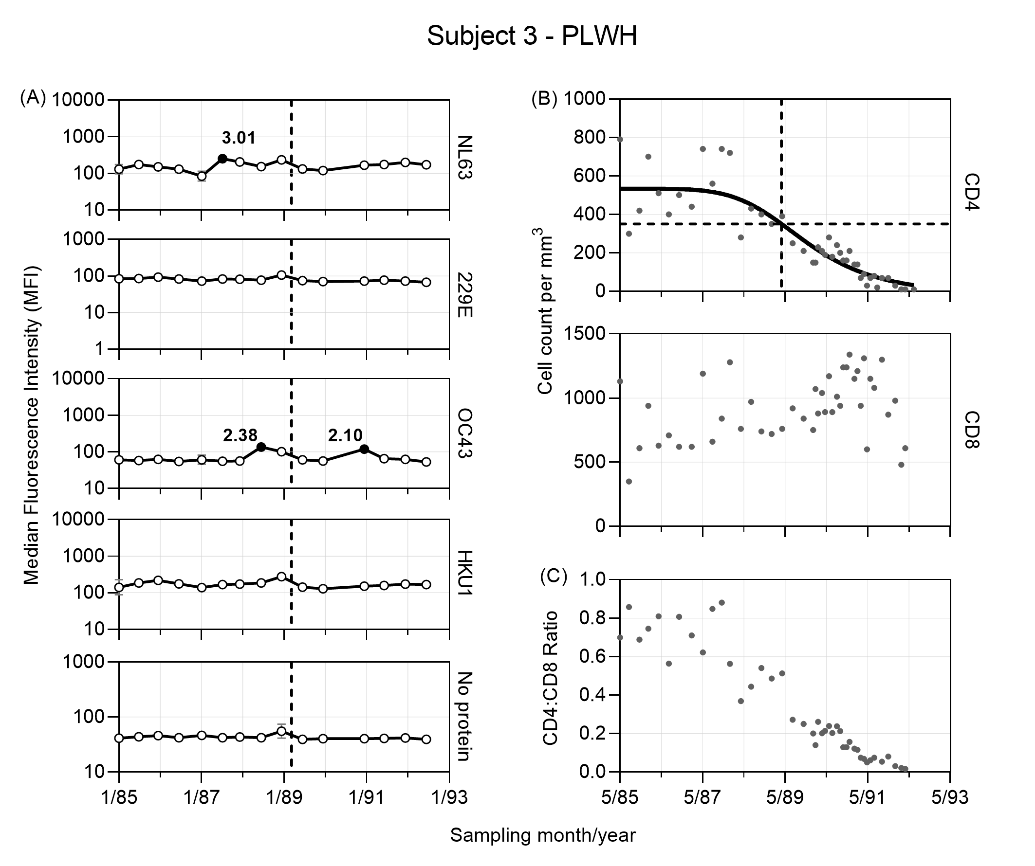
**

**S1 Fig (continued).**

**
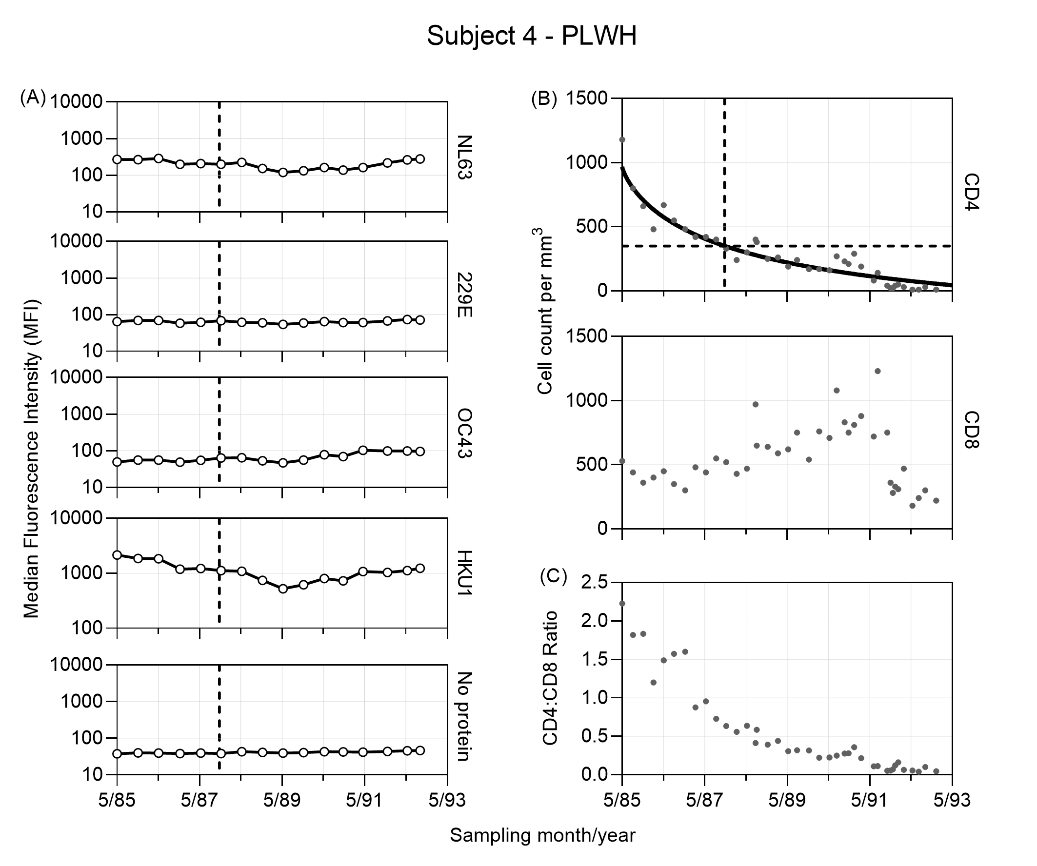
**

**
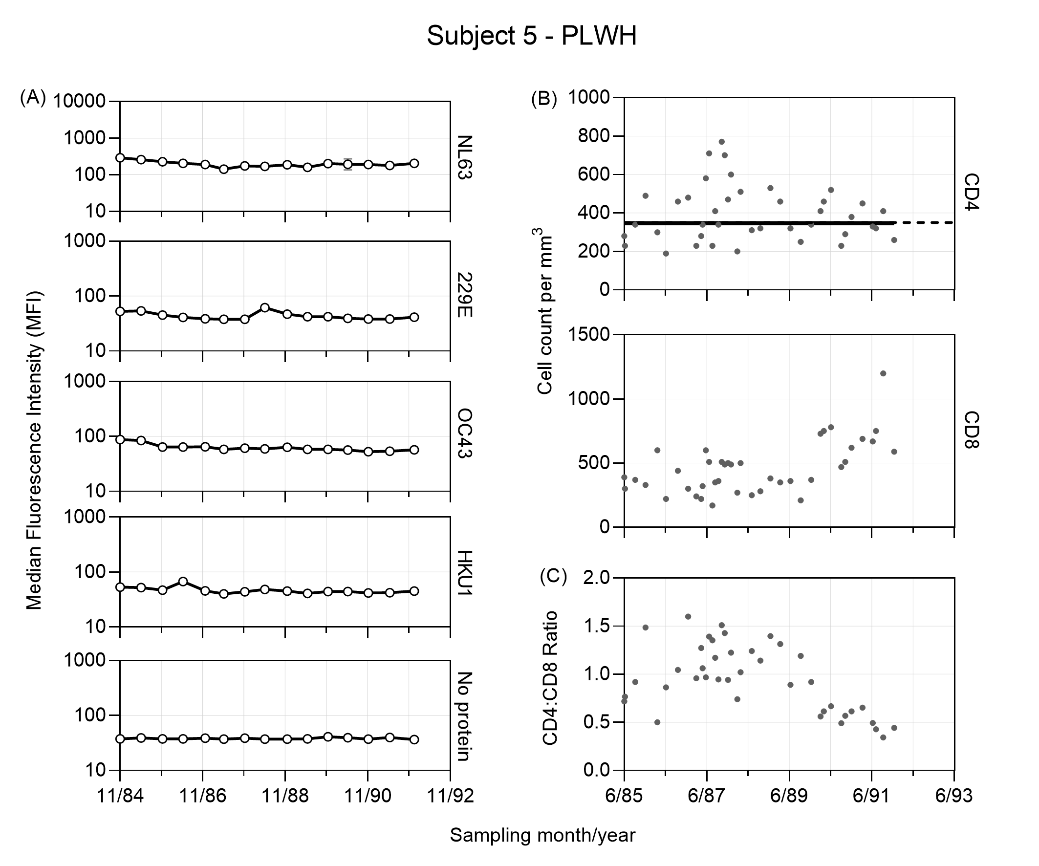
**

**S1 Fig (continued).**

**
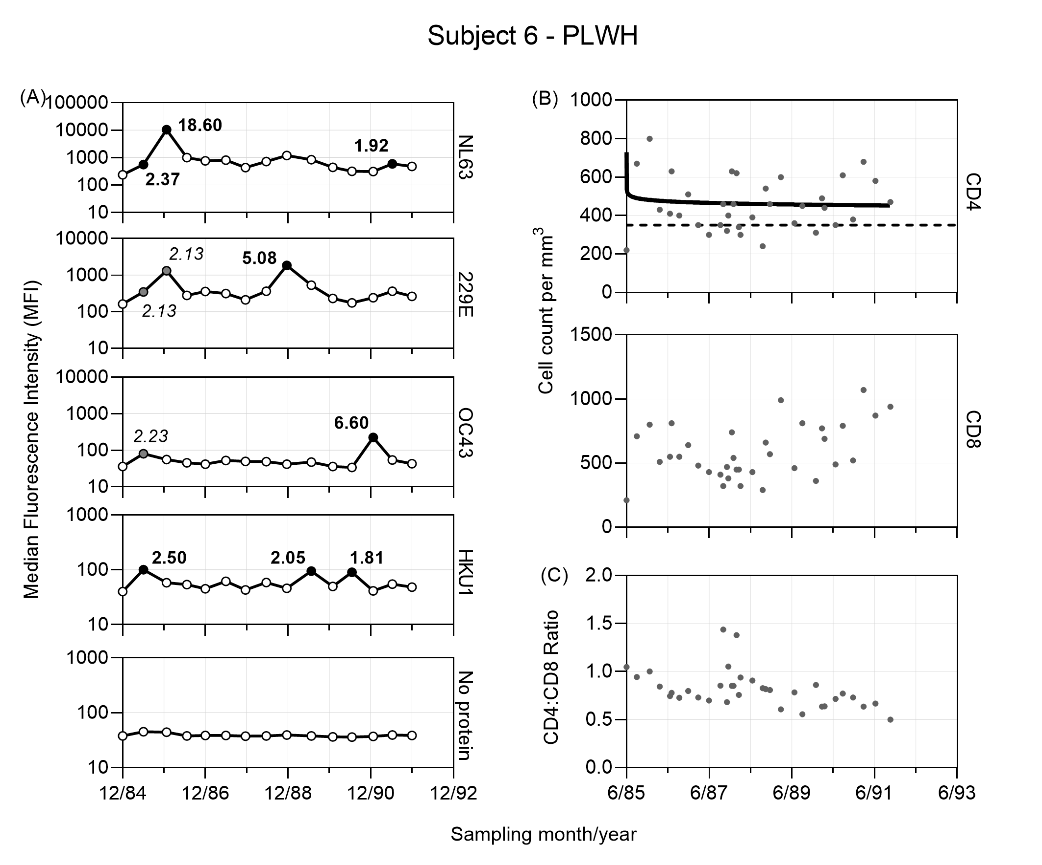
**

**
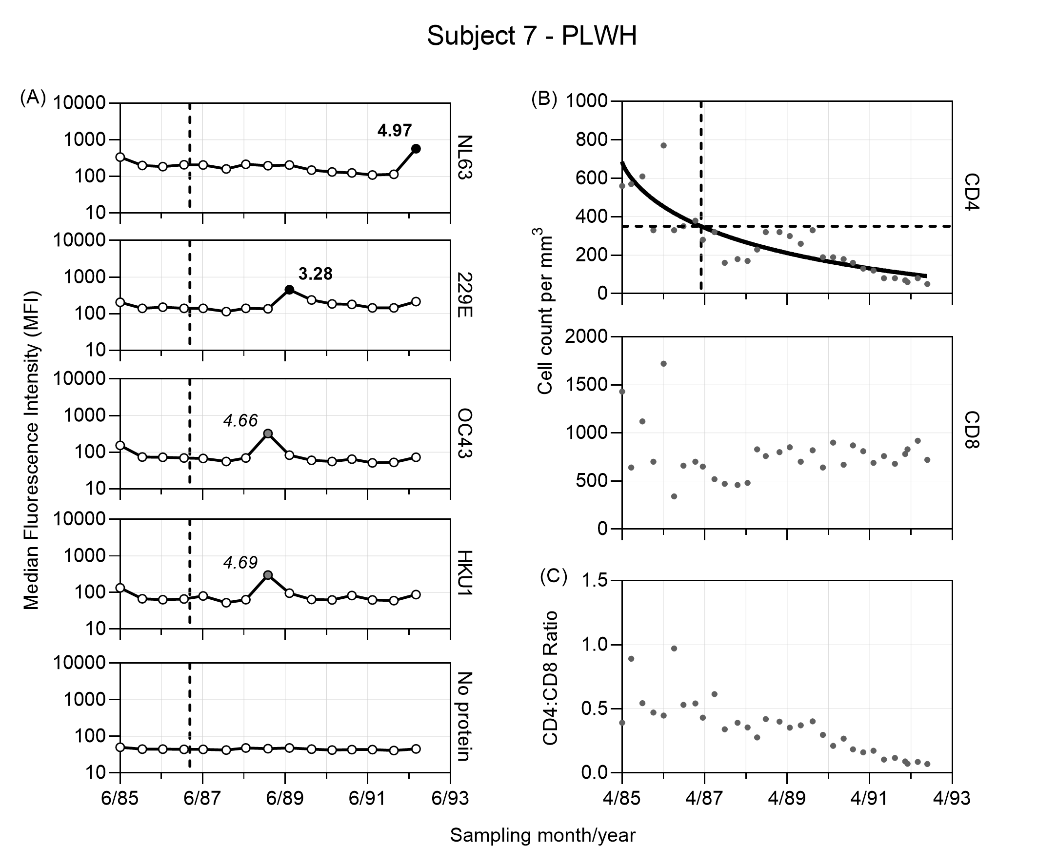
**

**S1 Fig (continued).**

**
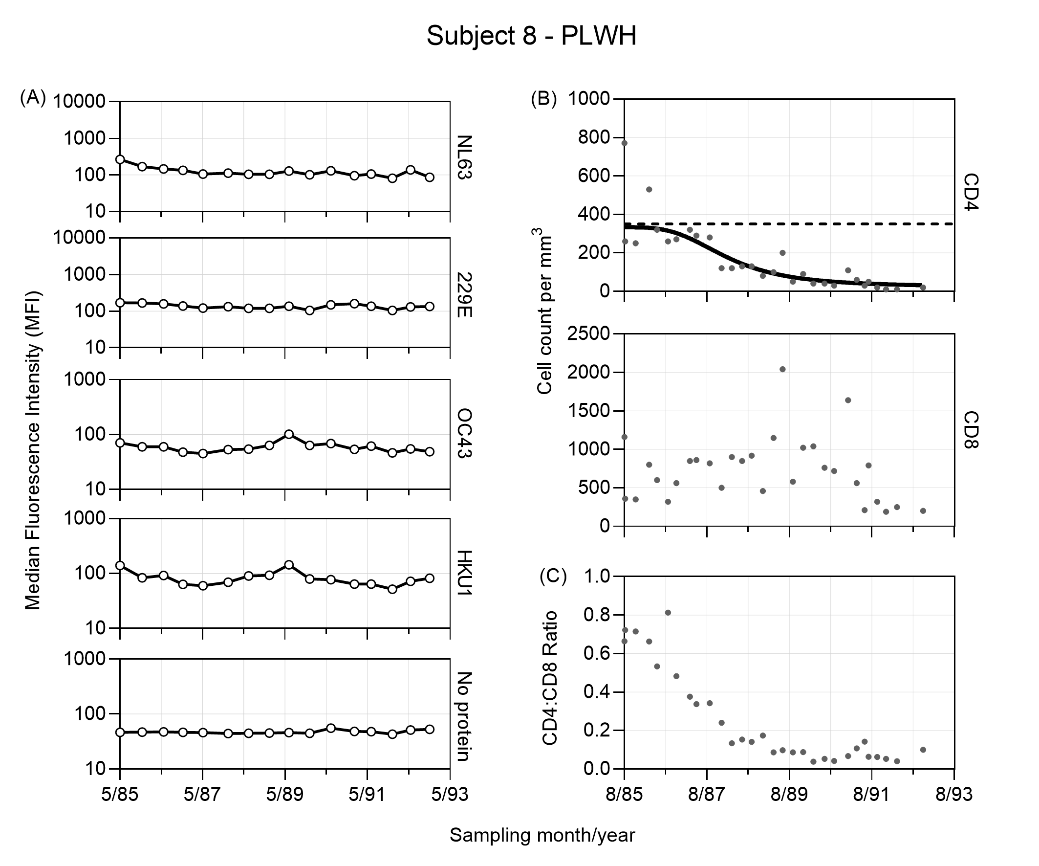
**

**
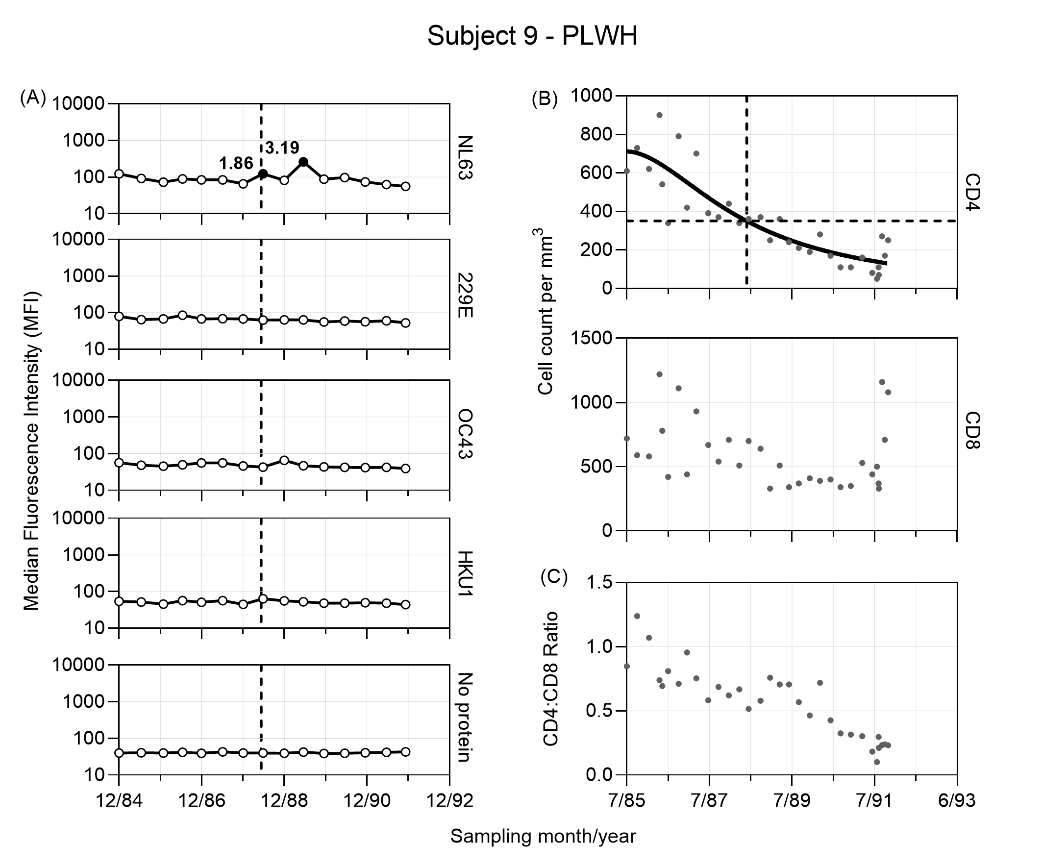
**

**S1 Fig (continued).**

**
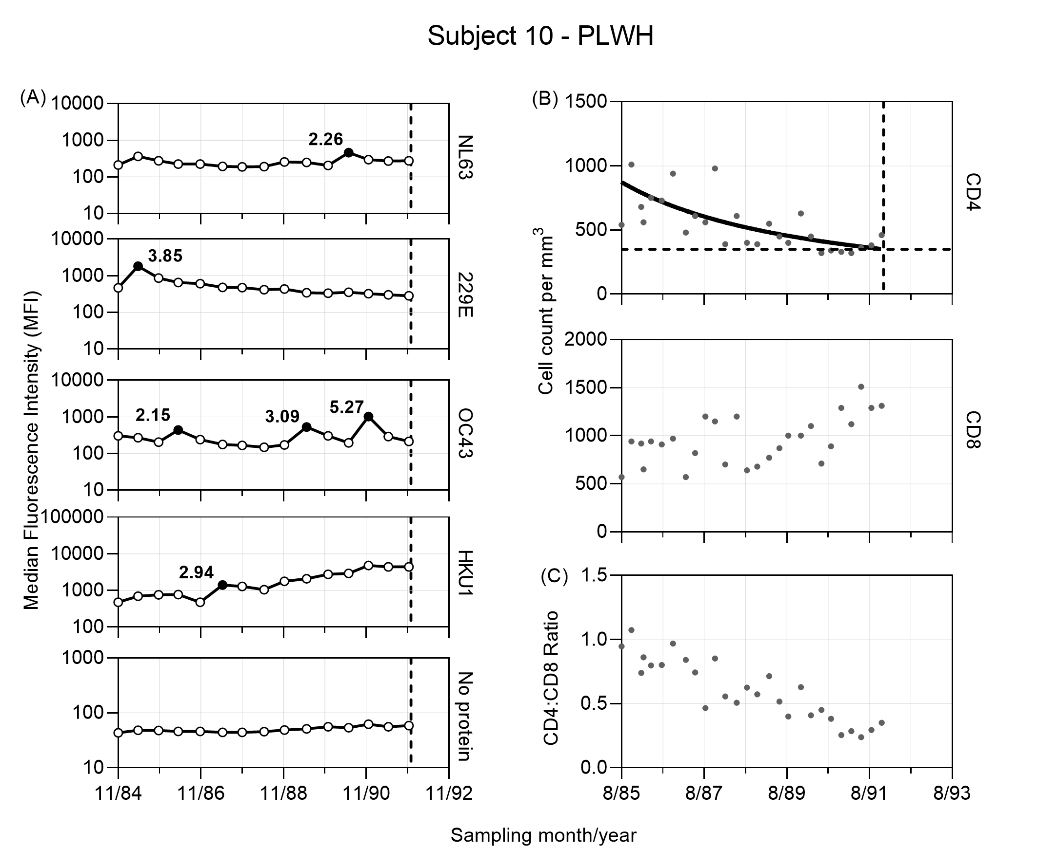
**

**
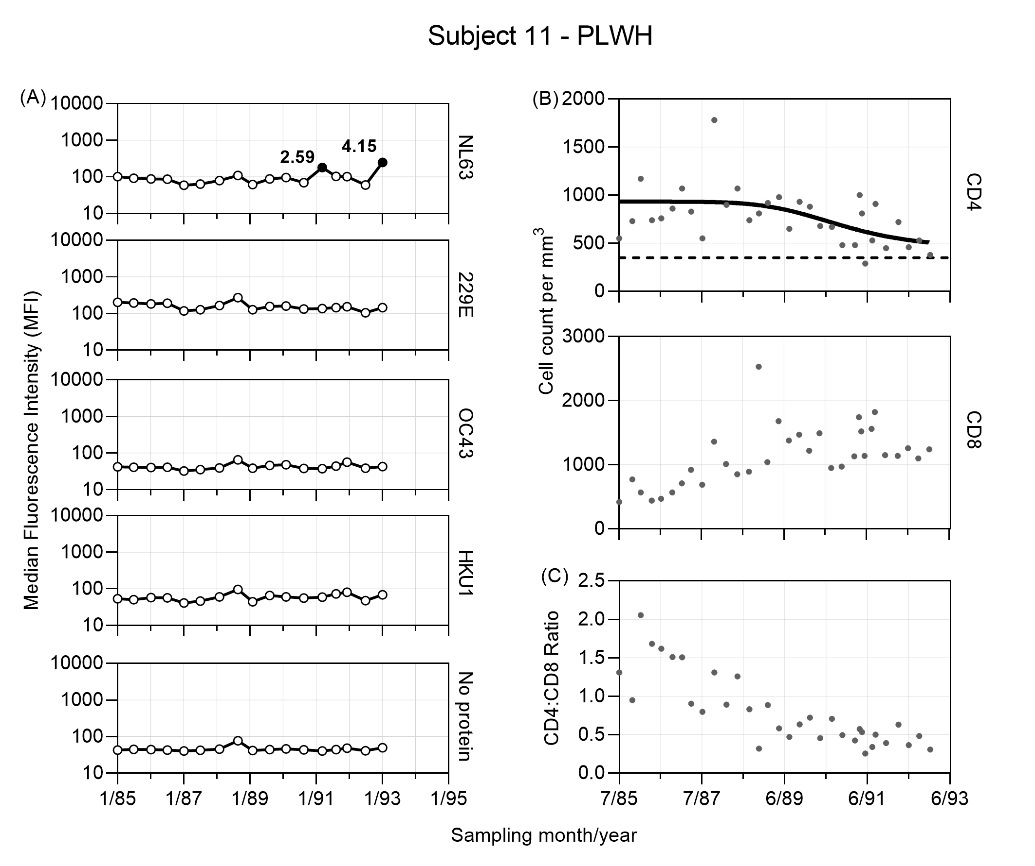
**

**S1 Fig (continued).**

**
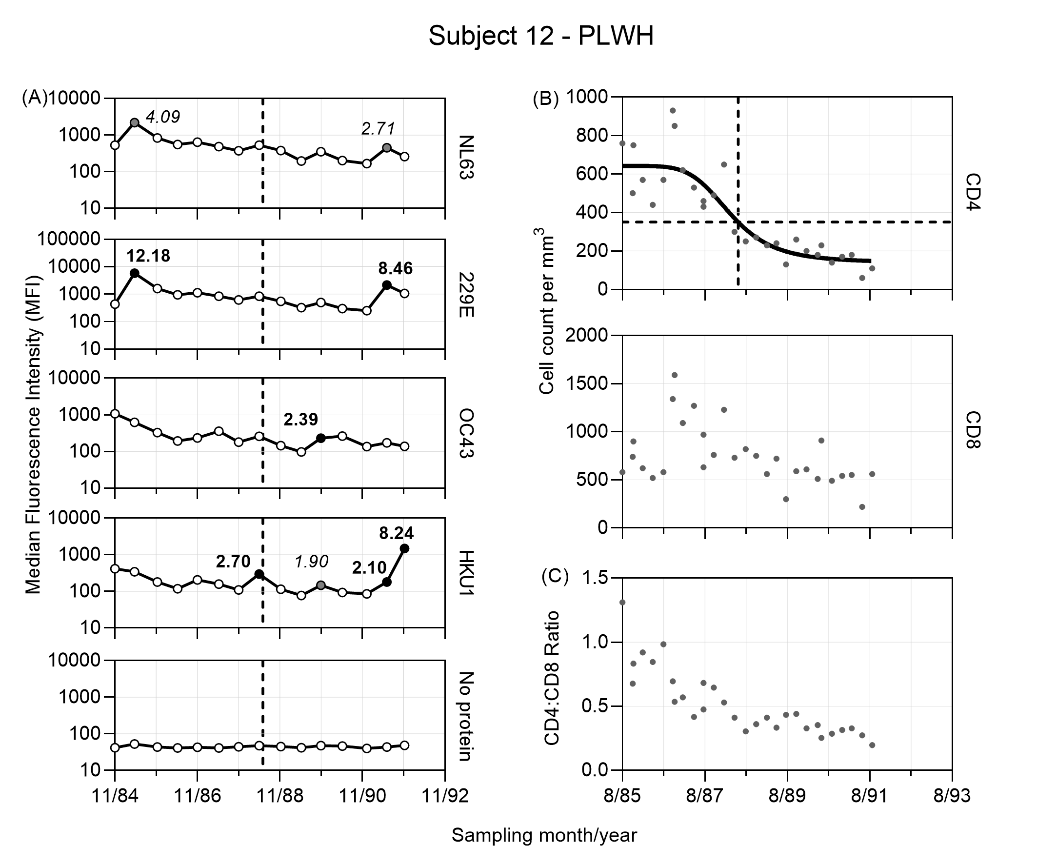
**

**
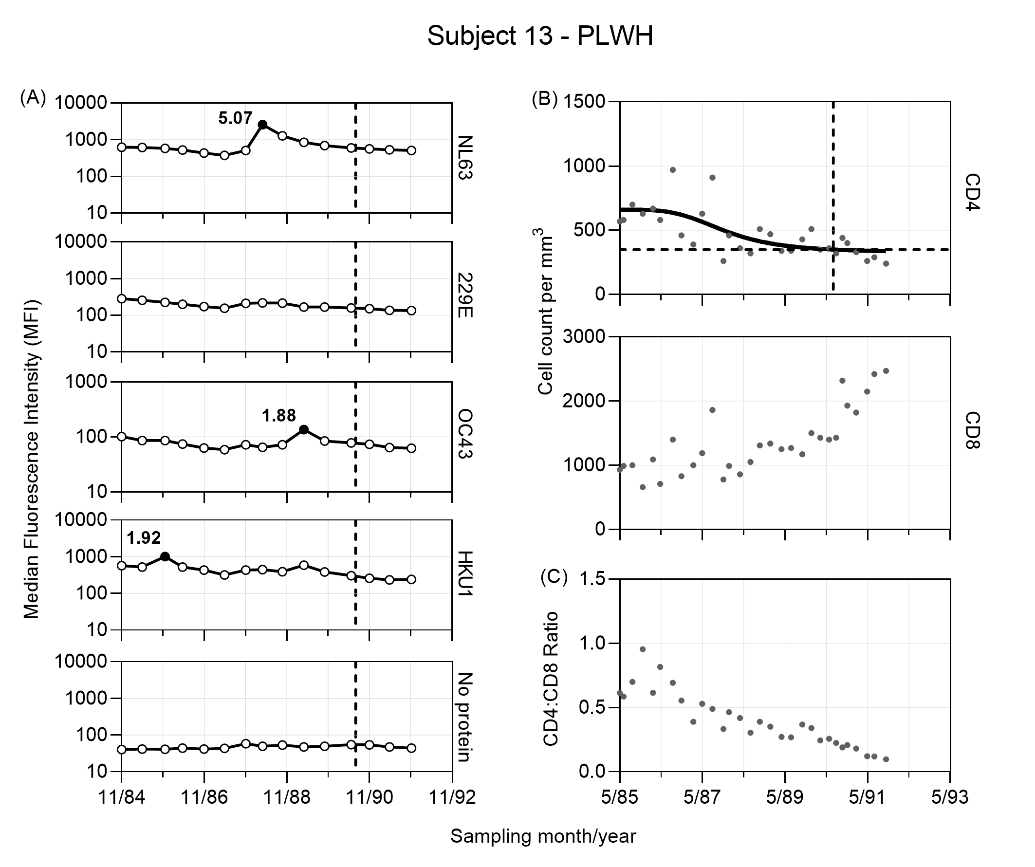
**

**S1 Fig (continued).**

**
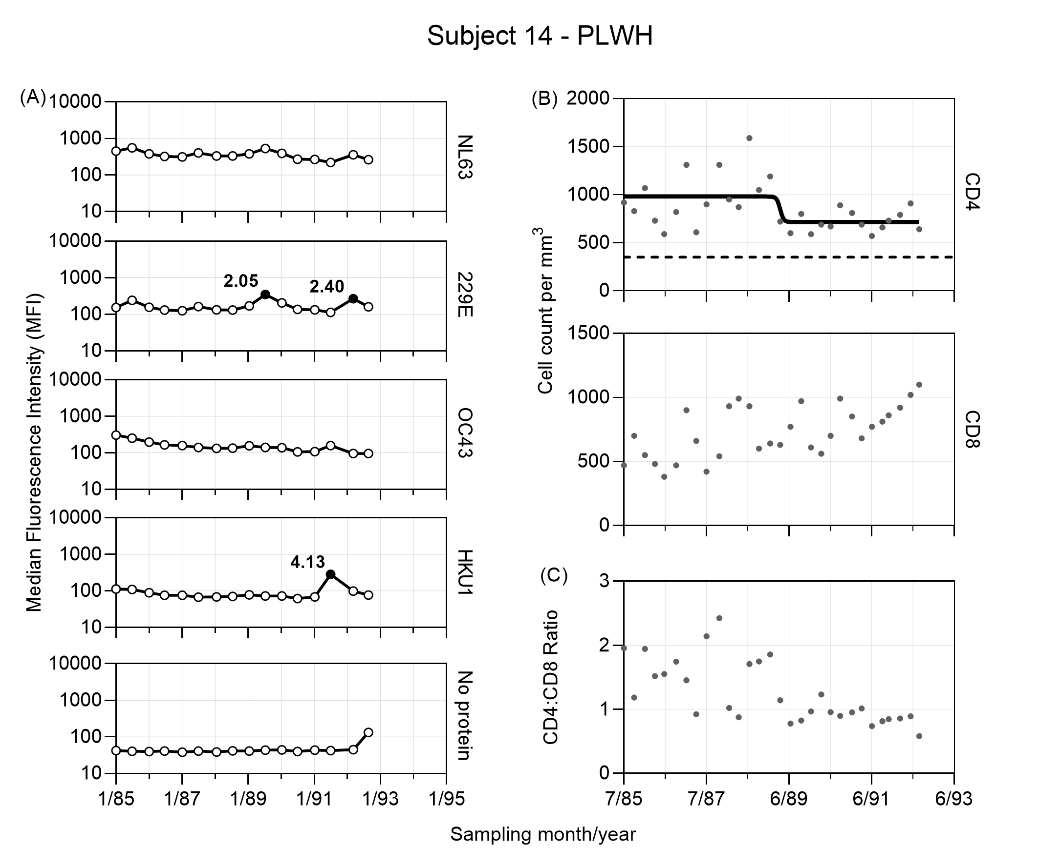
**

**
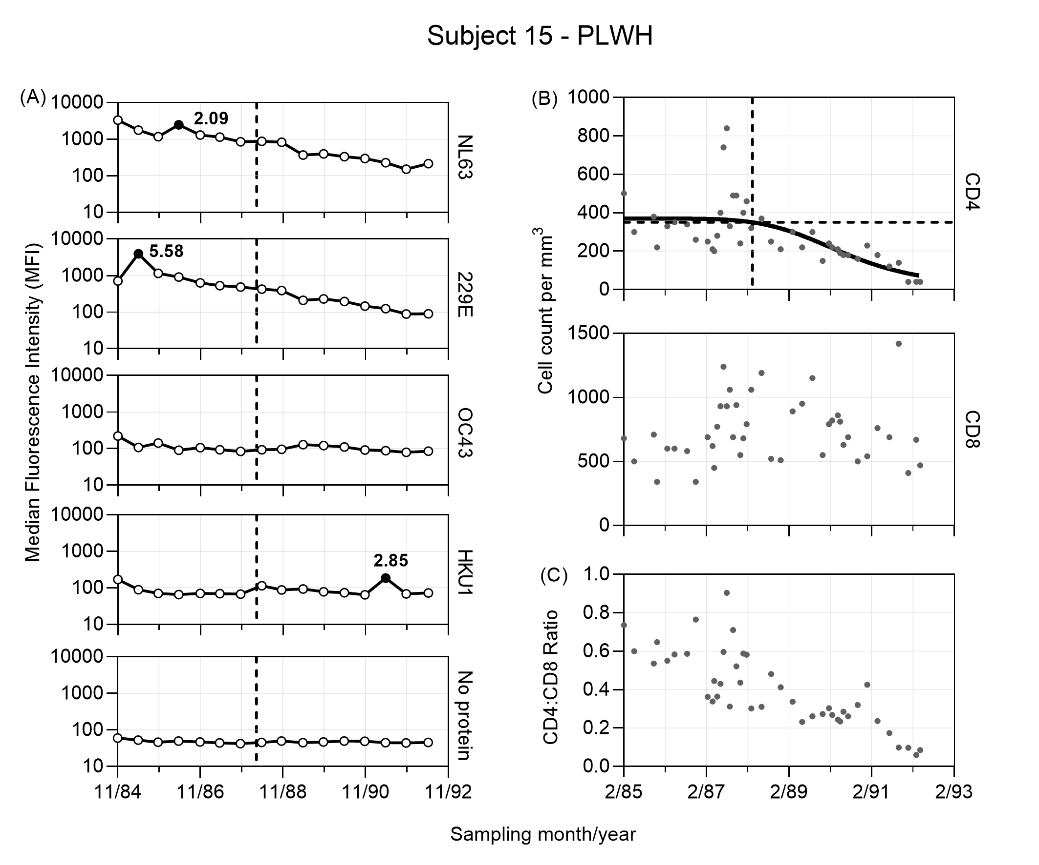
**

**S1 Fig (continued).**

**
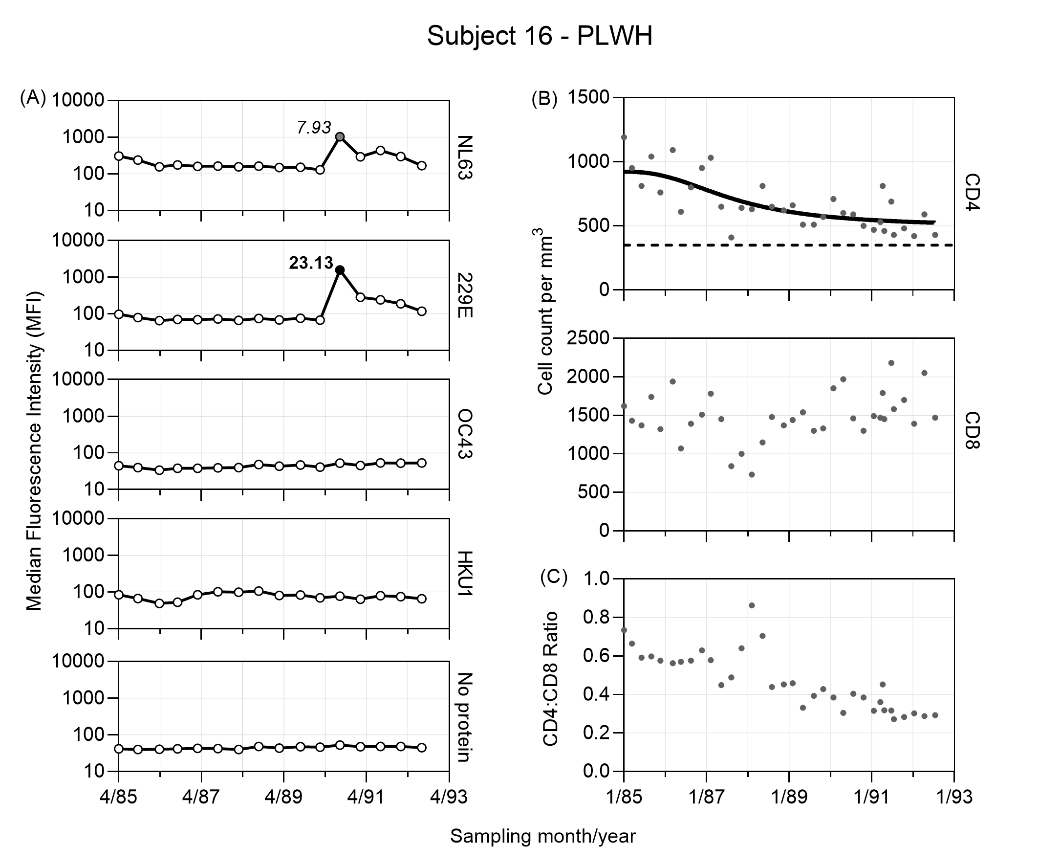
**

**
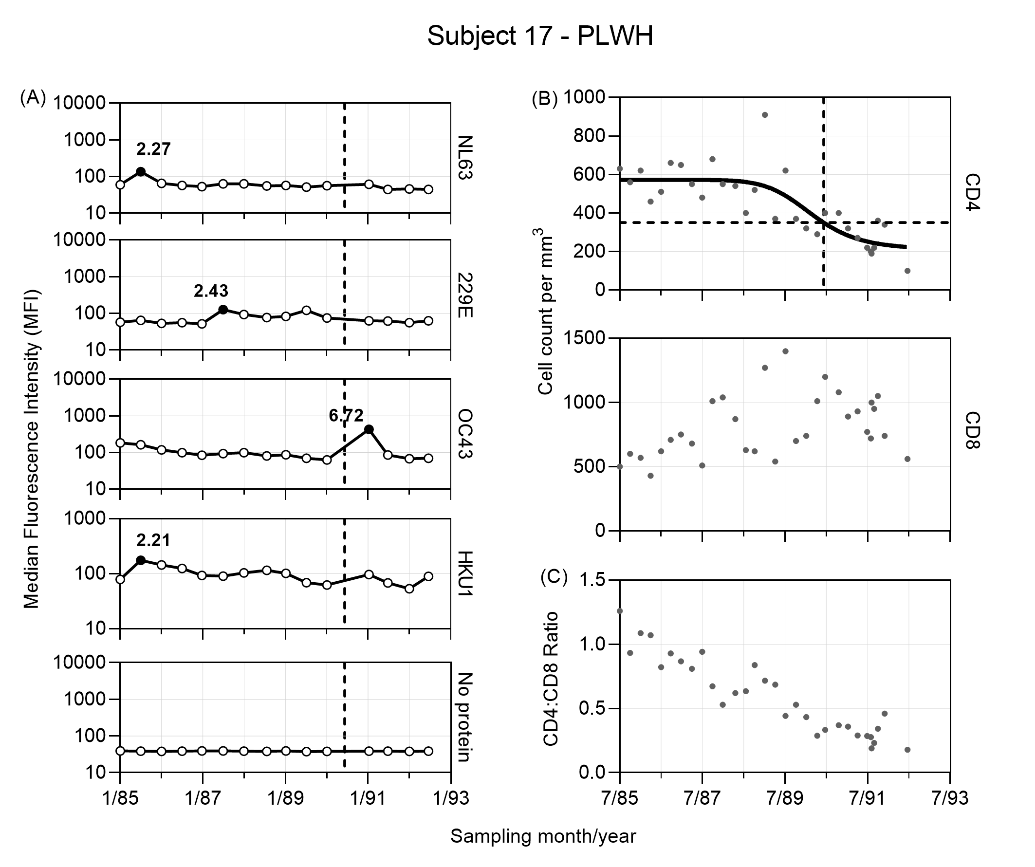
**

**S1 Fig (continued).**

**
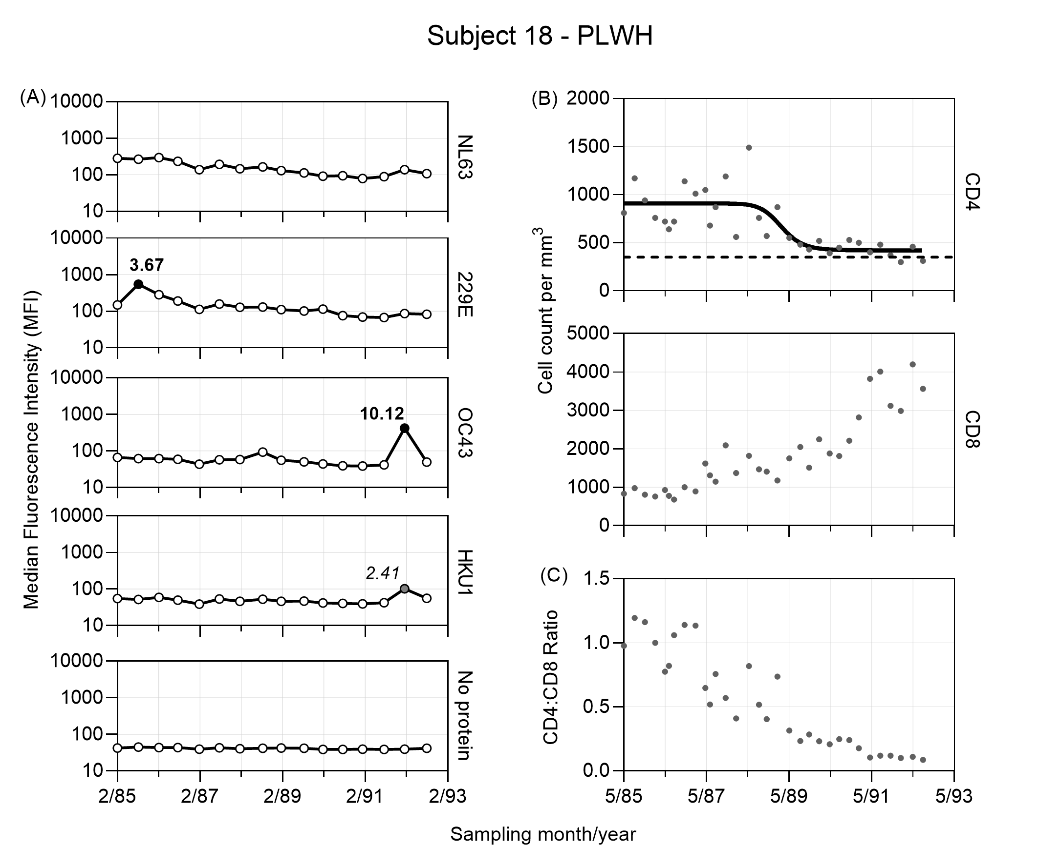

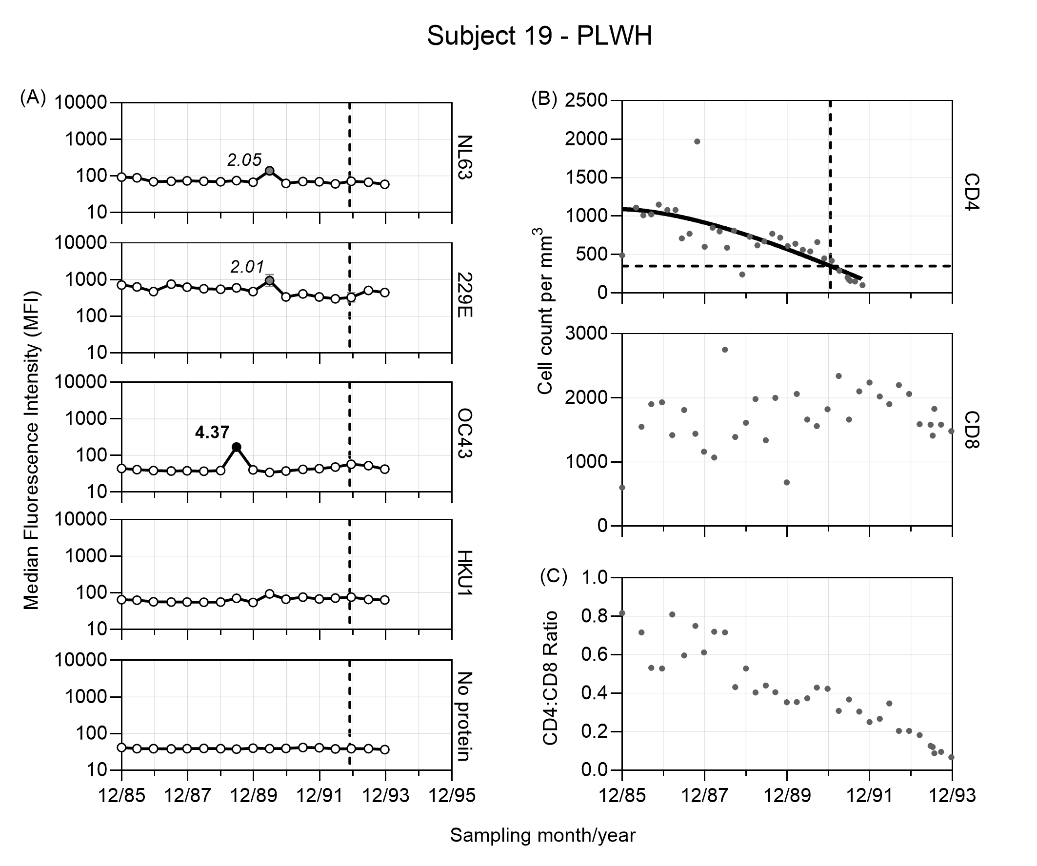
**

**S1 Fig (continued).**

**
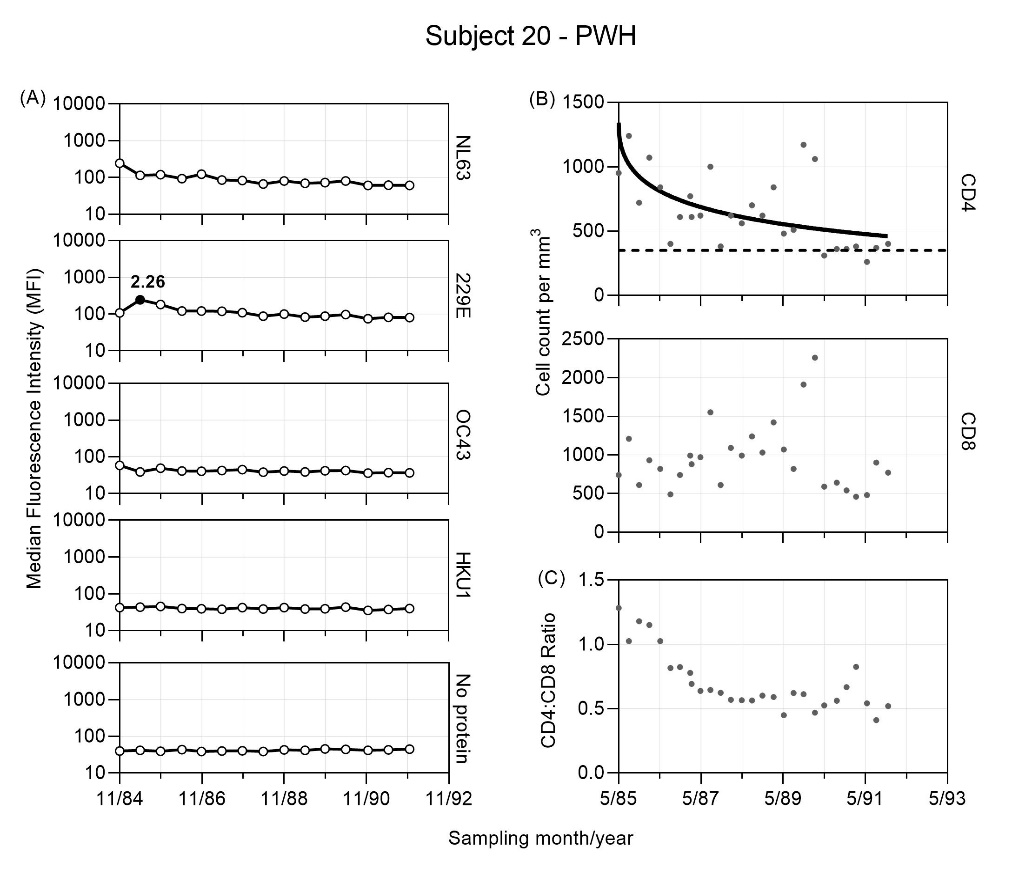
**

**
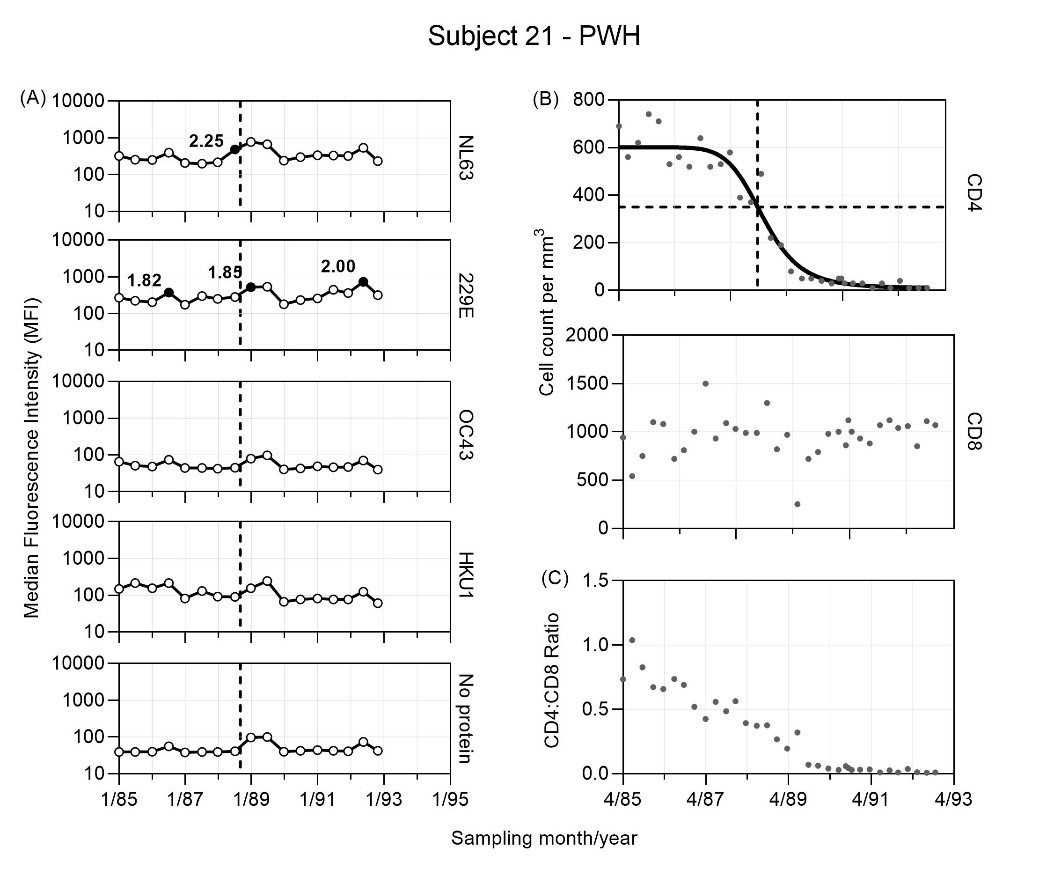
**

**S1 Fig (continued).**

**
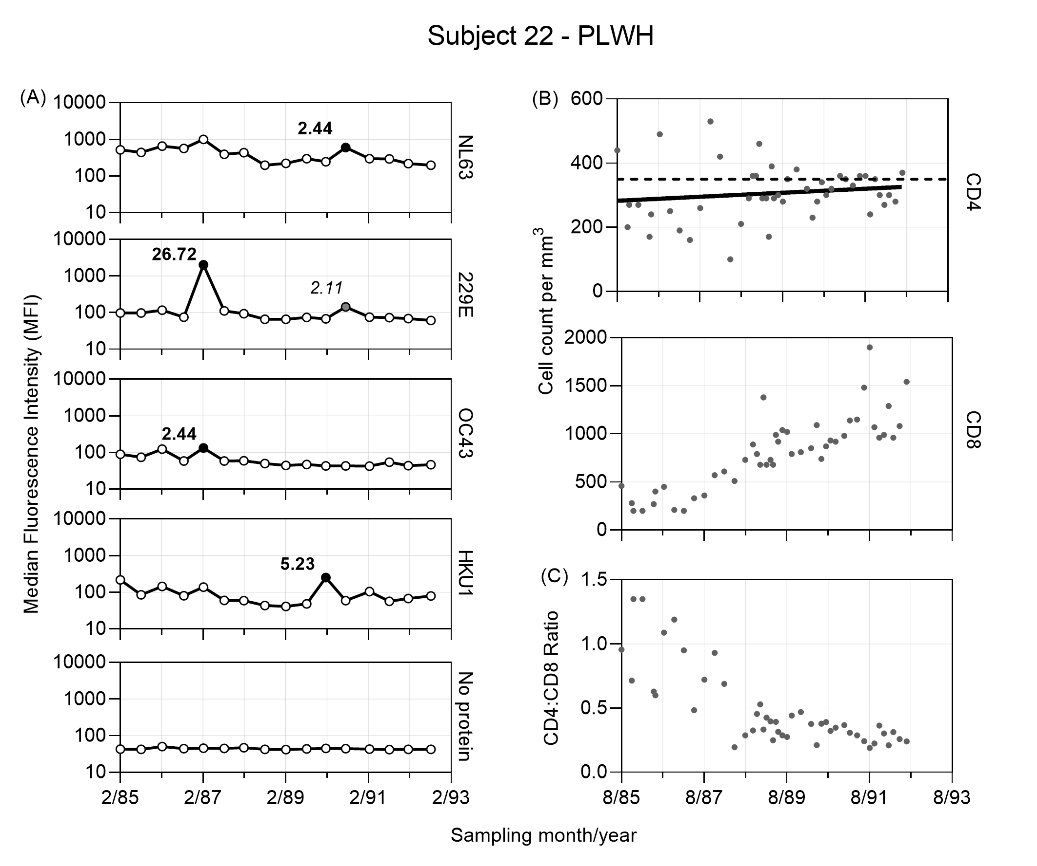

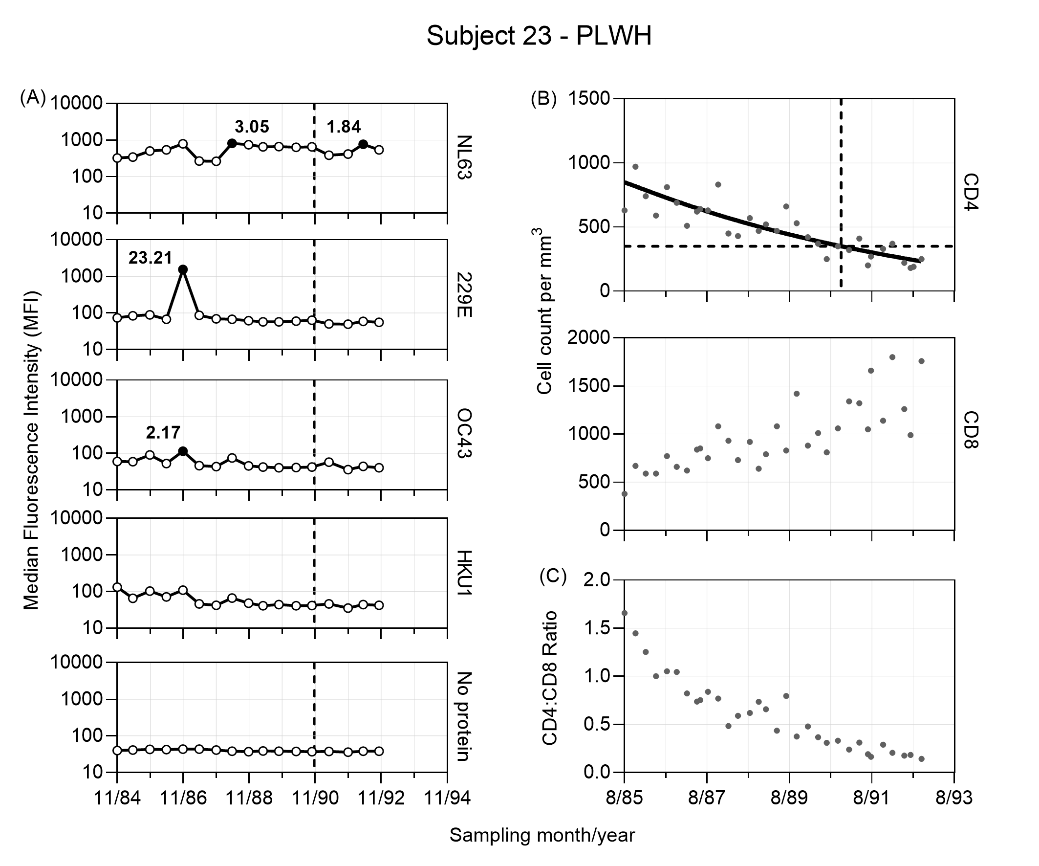
**

**S1 Fig (continued).**


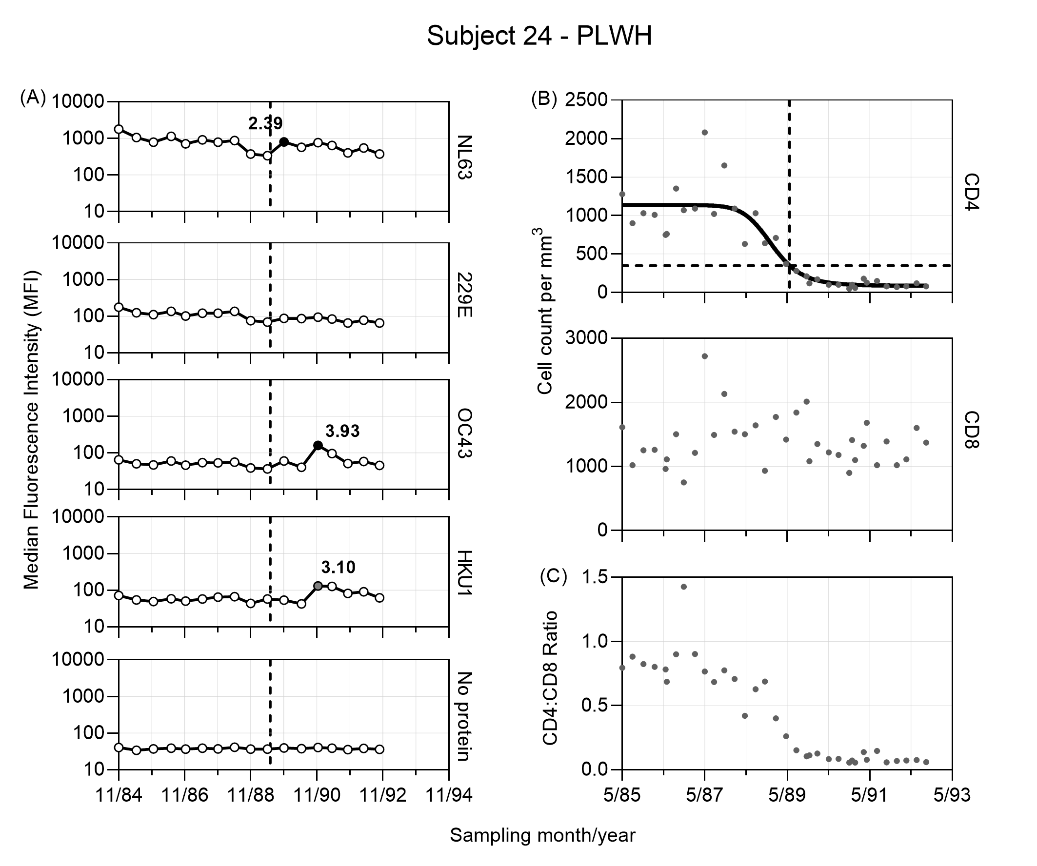


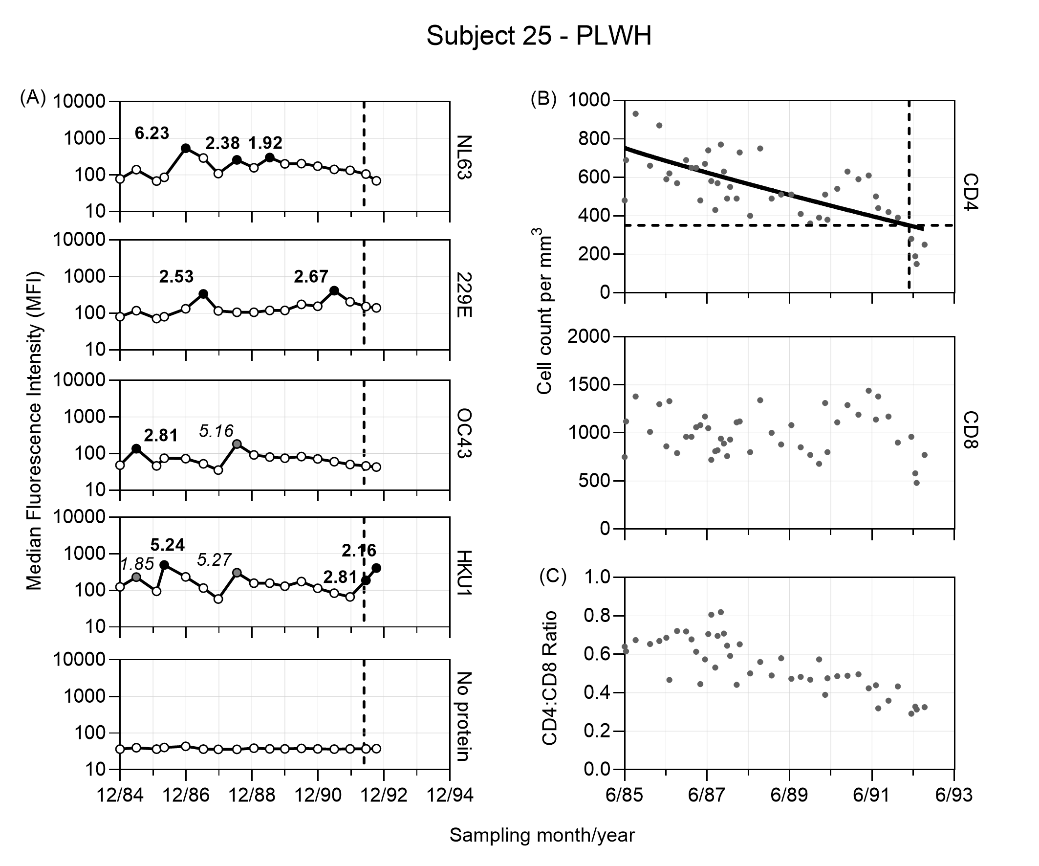

Supplement: S1 Fig — (DOCX) [file pgph.0004610.s009.docx]
