# Supplementary material for: Untreated HIV-1 infection and low CD4+ T cell counts and their effect on endemic human coronavirus (re)infection
Source: PLOS Glob Public Health. 2025 Jun 18;5(6):e0004610. doi: 10.1371/journal.pgph.0004610 (PMC12176178; doi:10.1371/journal.pgph.0004610)
Supplement: S2 Fig — (DOCX) [file pgph.0004610.s010.docx]

**Supplementary Material**

**Untreated HIV-1 infection and low CD4^+^ T cell counts and their effect on endemic HCoV (re)-infection**

Ferdyansyah Sechan, Anne W. M. van den Hurk, T. Sonia Boender, Maria Prins, Amy Matser, Margreet Bakker, Neeltje A. Kootstra, and Lia van der Hoek

**
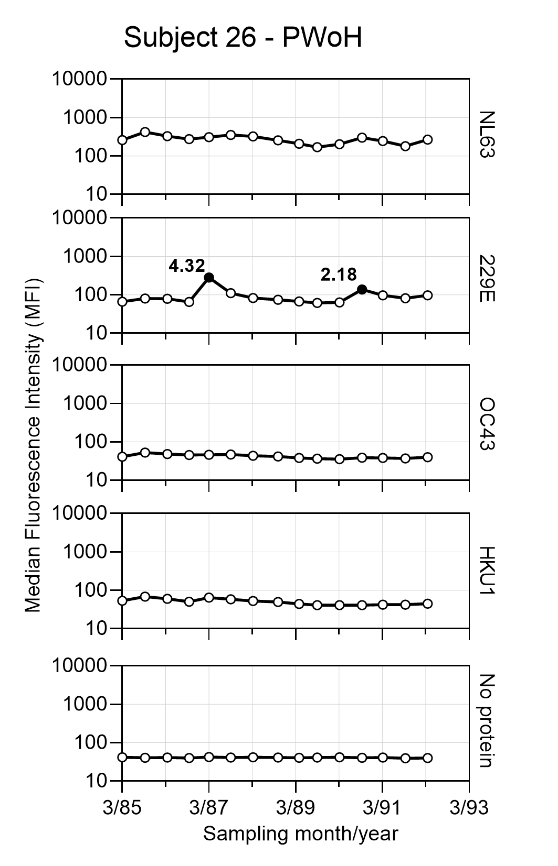
**

**S2 Fig. Antibody dynamics for PWoH (subject 26-50); see next pages for Subjects 27-50.**

Data is presented as geometric mean ± SD of two technical replicates for each serum sample in median fluorescence intensity (MFI). White dots: fold-change < 1.8. Black dots: fold change ≥ 1.80 as the indication of infection with fold change values given in black and bold above the data points. Gray dots: fold change ≥ 1.80 but not counted as infection due to either being cross-reaction (within-genus fold-change difference > 10%) or no difference could be made (within-genus fold-change difference ≤ 10%); fold-change values are given in black and italic above the data points.

**S2 Fig (continued).**

**
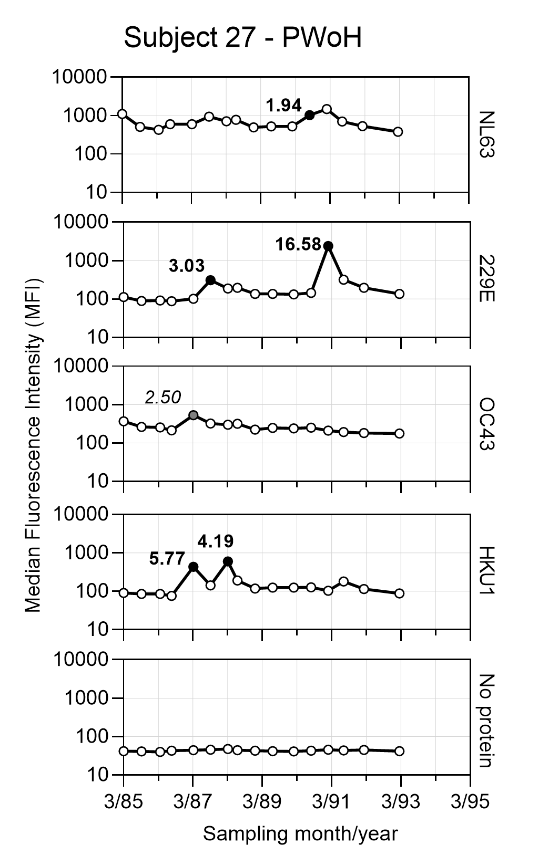

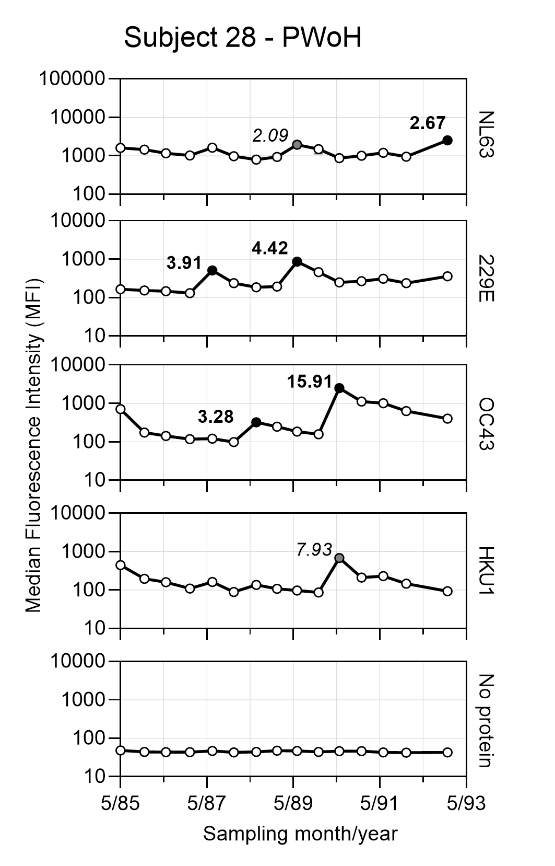

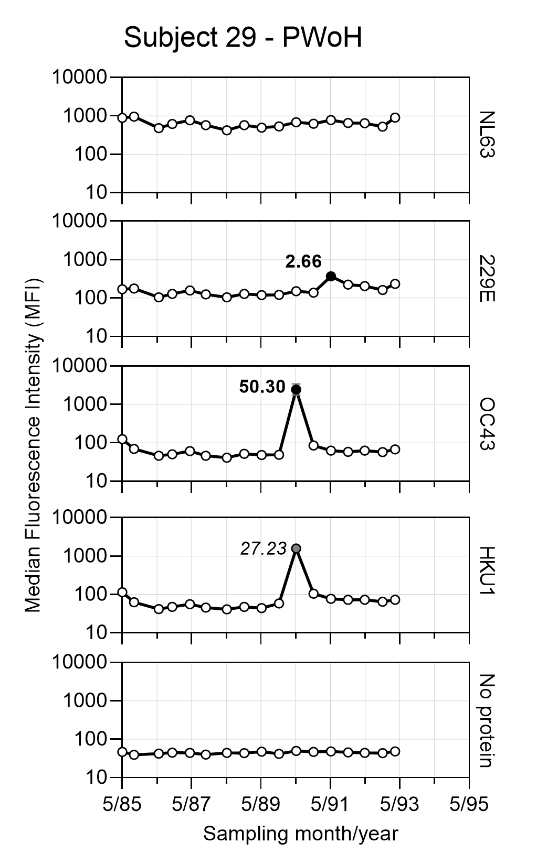

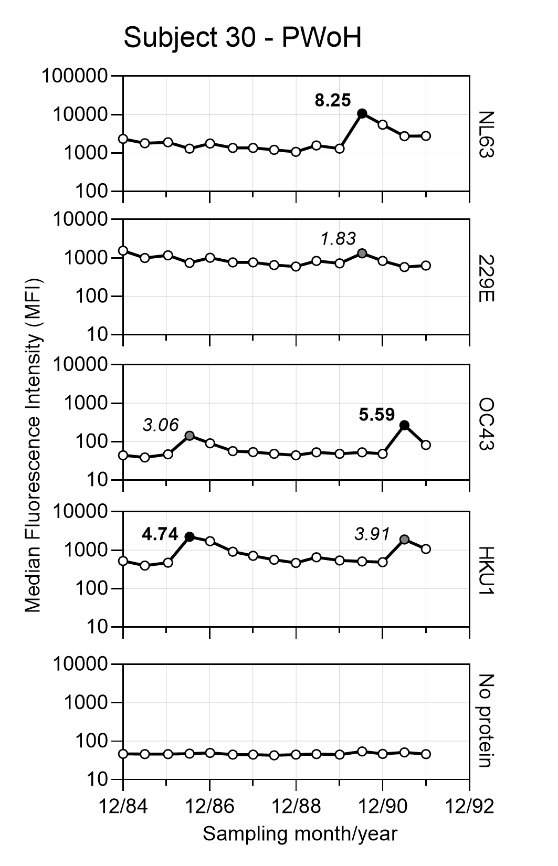
**

**S2 Fig (continued).**

**
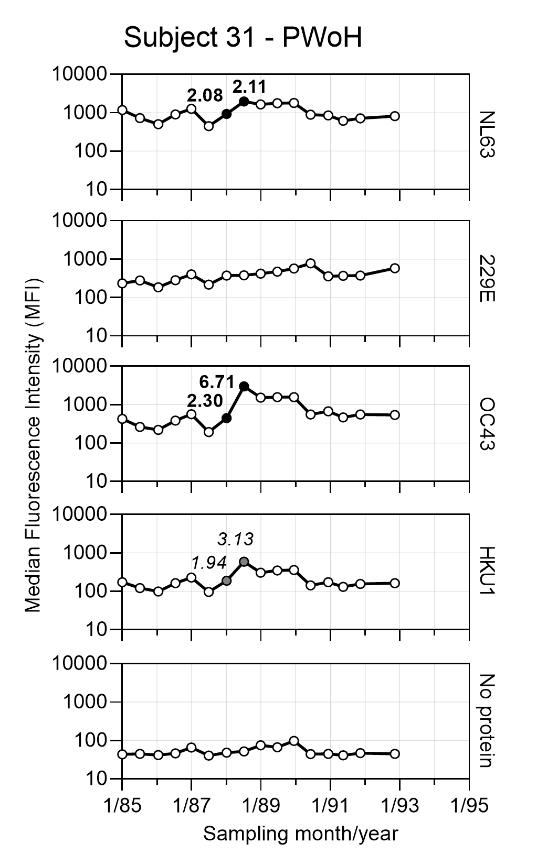

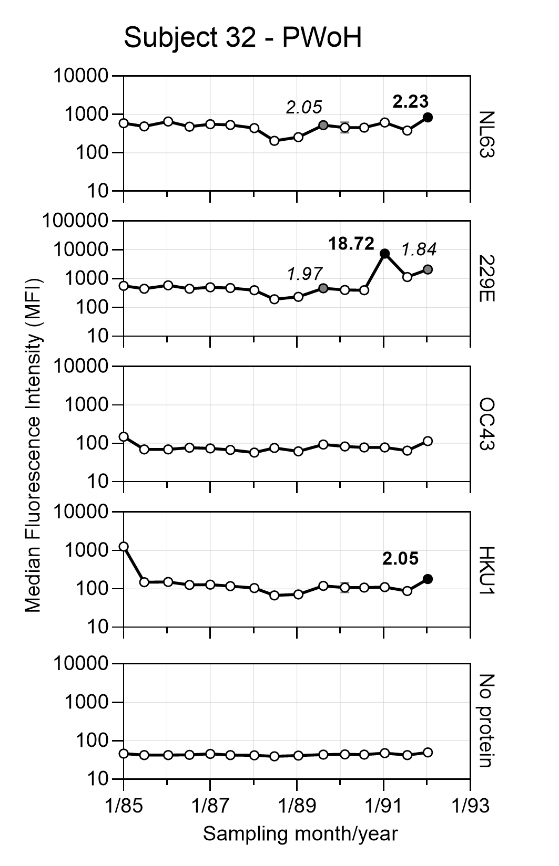

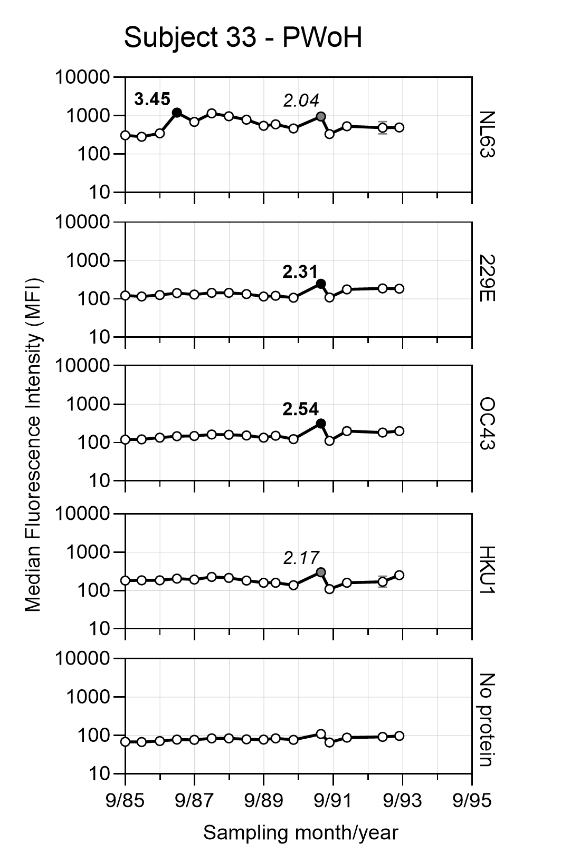

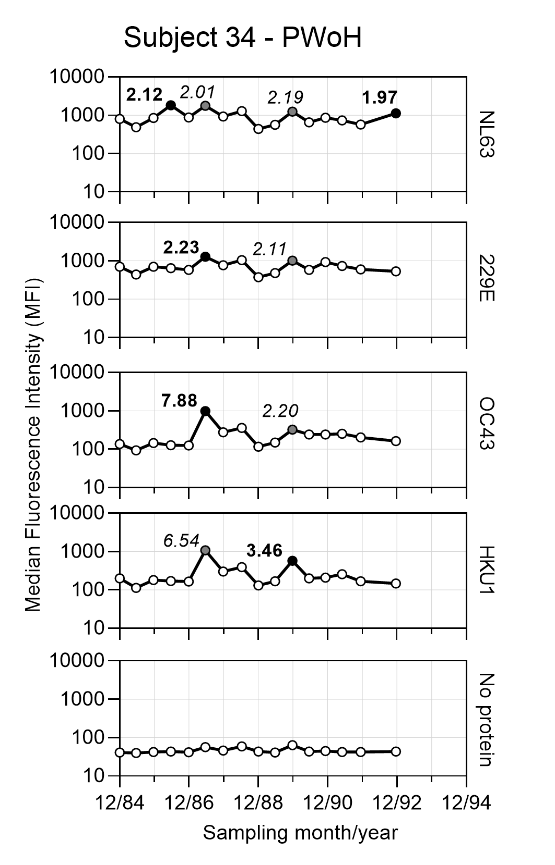
 S2 Fig (continued).**

**
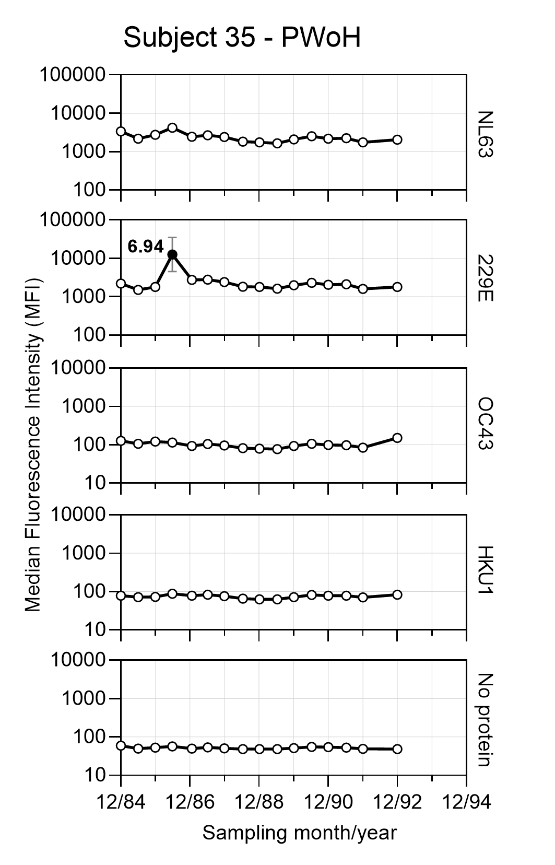

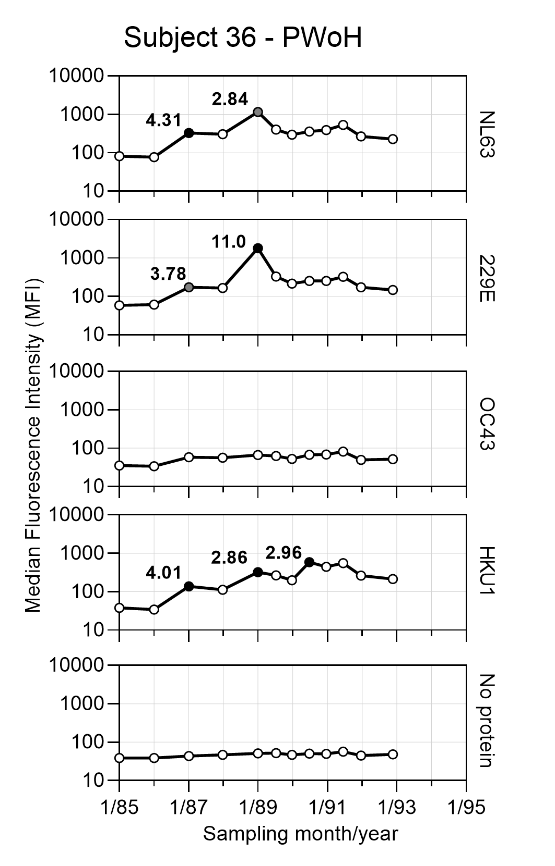

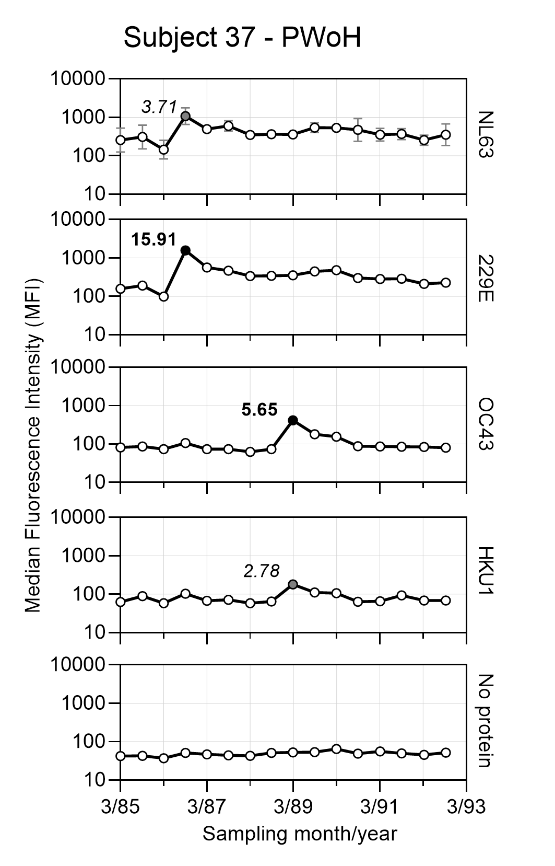

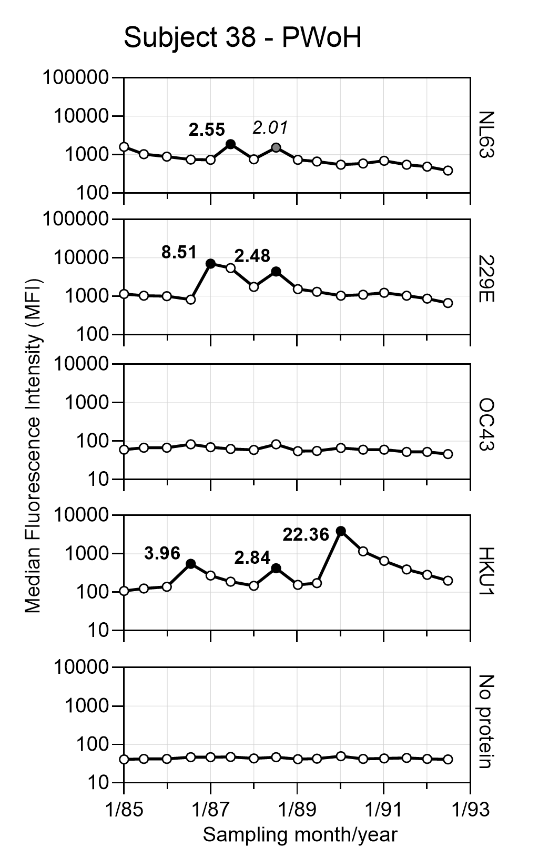
**

**S2 Fig (continued).**

**
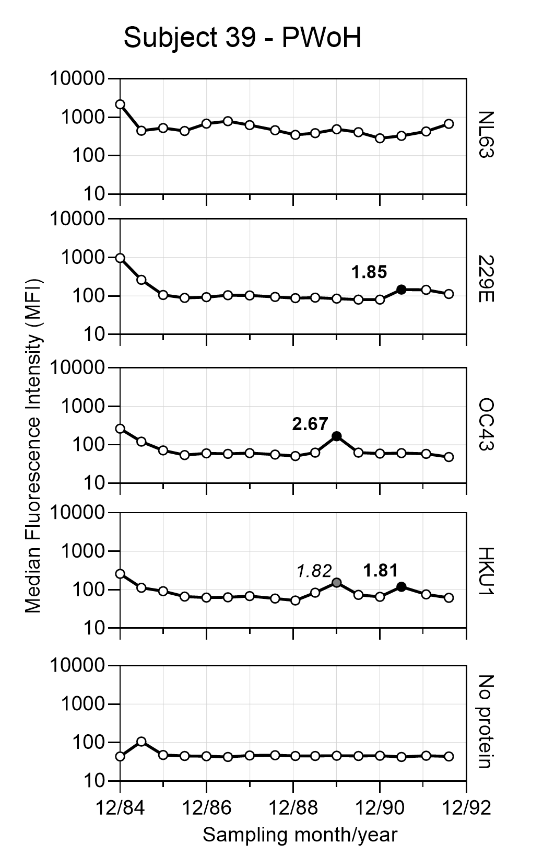

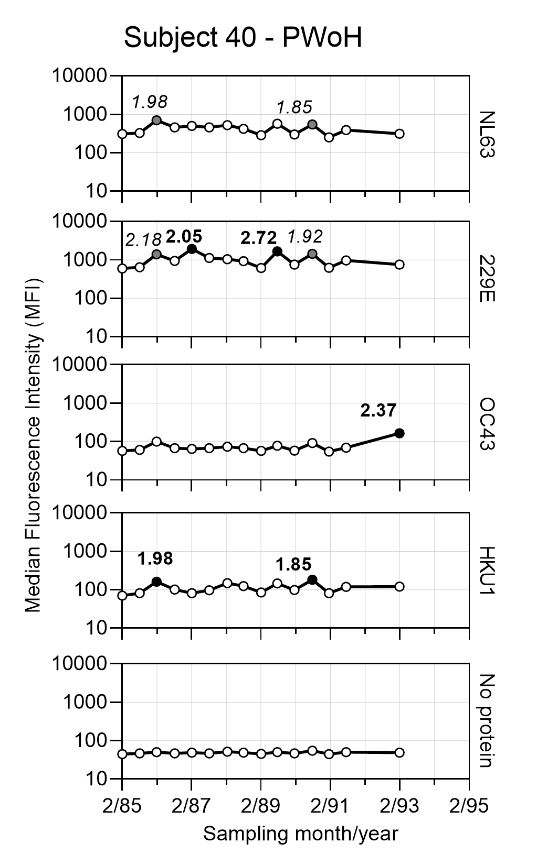

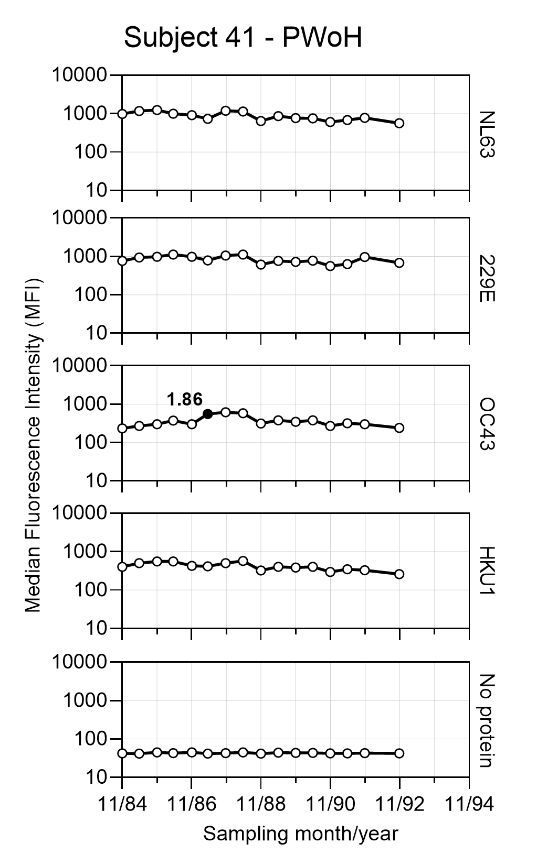

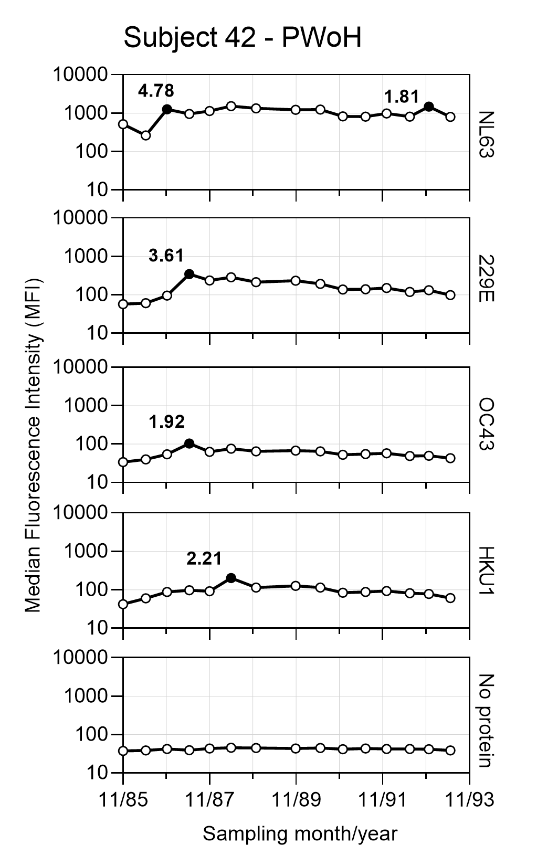
**

**S2 Fig (continued).**

**
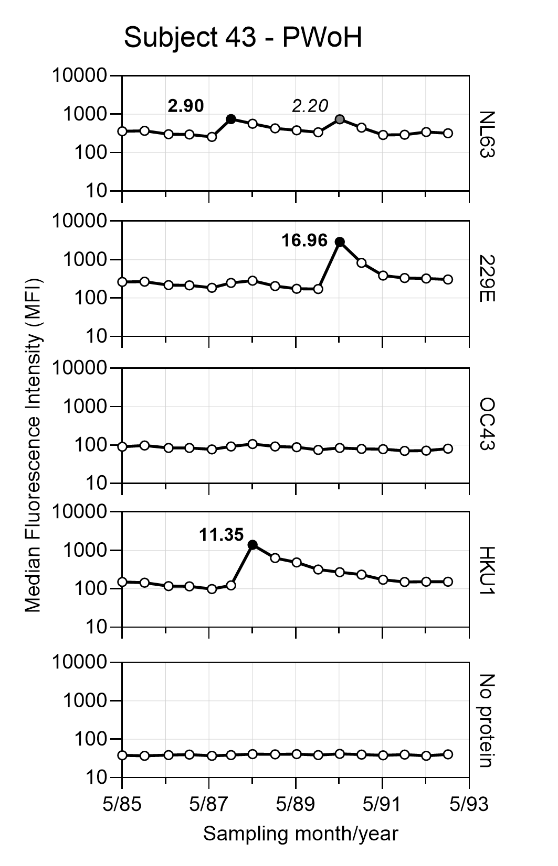

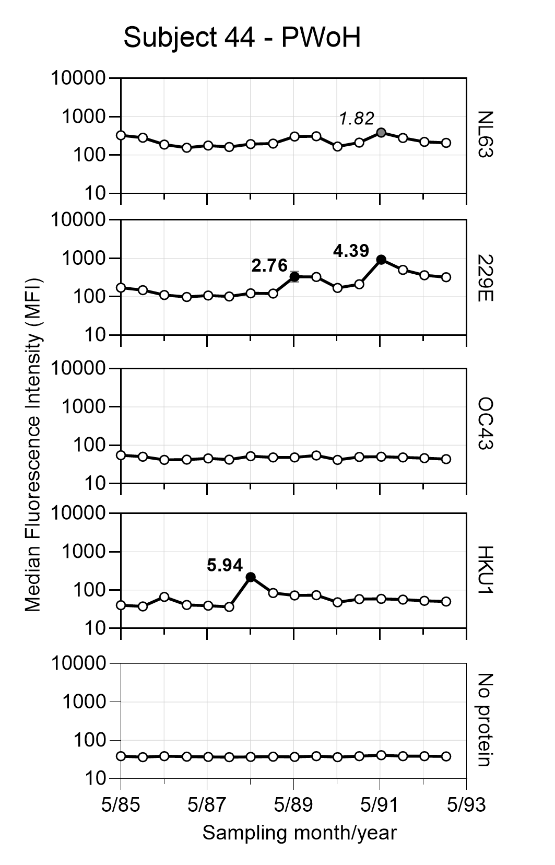

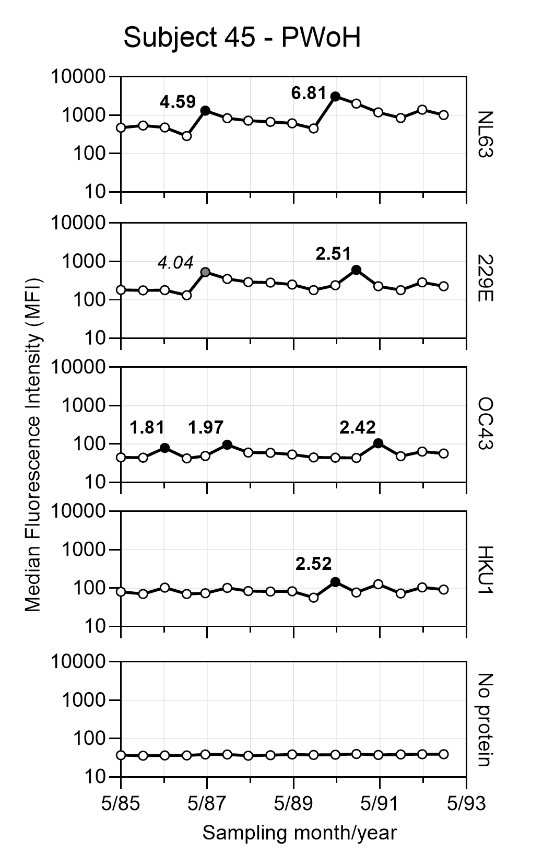

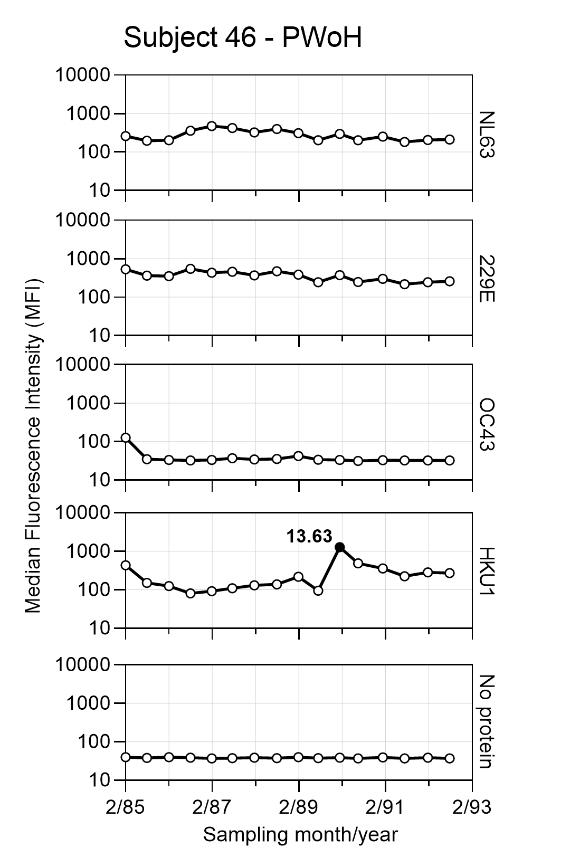
**

**S2 Fig (continued).**

**
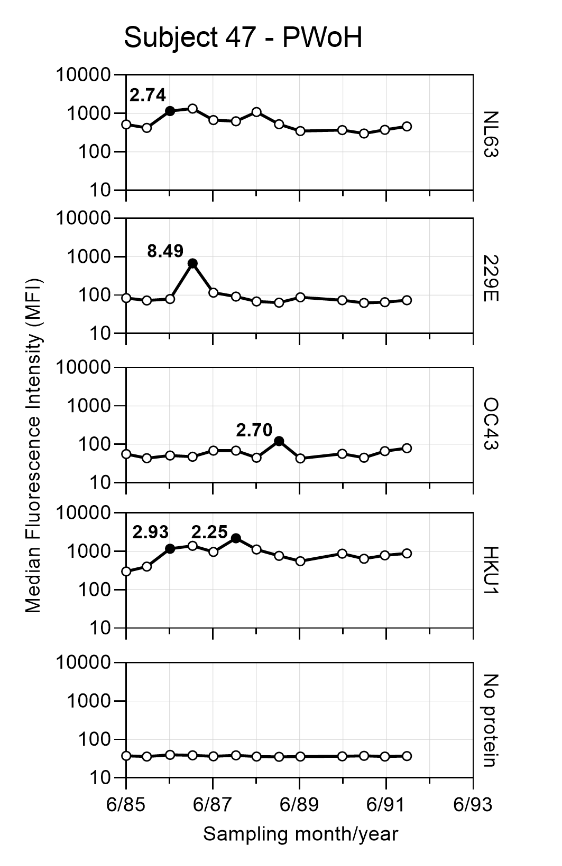

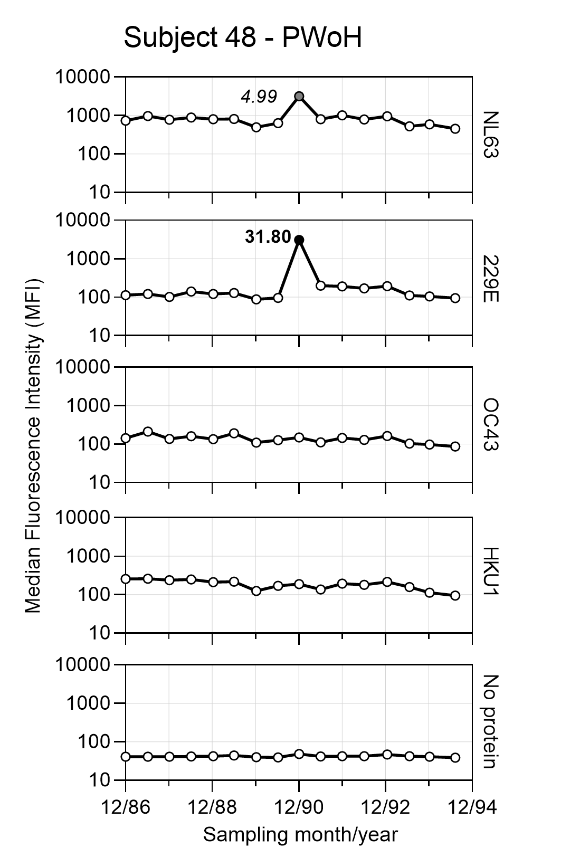

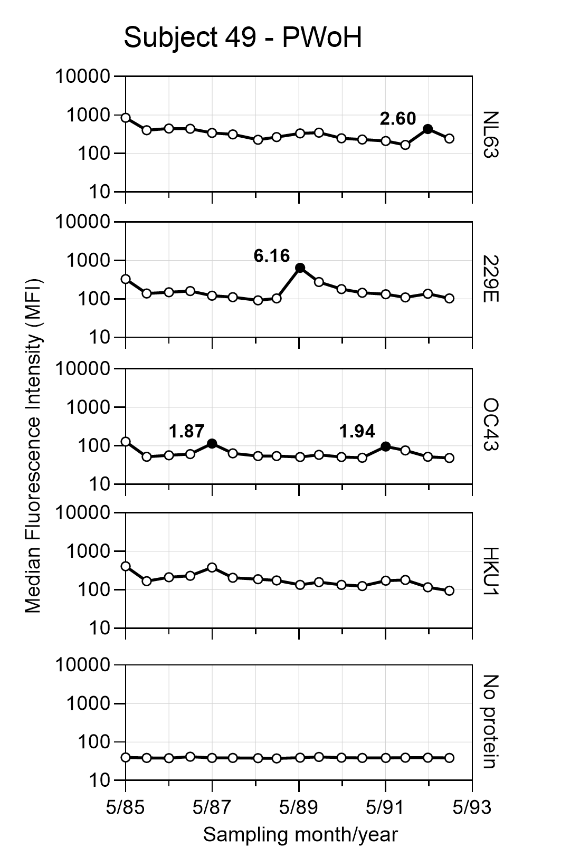

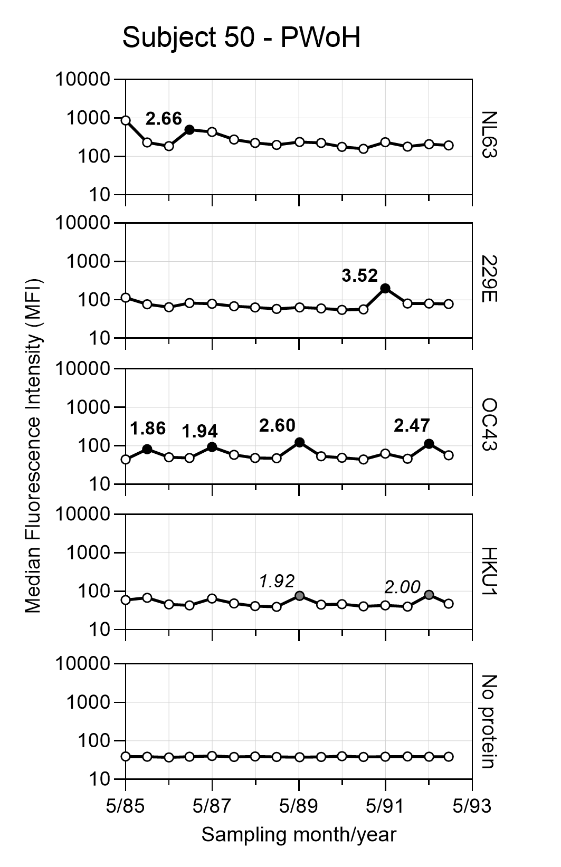
**
