# Supplementary material for: Untreated HIV-1 infection and low CD4+ T cell counts and their effect on endemic human coronavirus (re)infection
Source: PLOS Glob Public Health. 2025 Jun 18;5(6):e0004610. doi: 10.1371/journal.pgph.0004610 (PMC12176178; doi:10.1371/journal.pgph.0004610)
Supplement: S3 Fig — (DOCX) [file pgph.0004610.s011.docx]

**Supplementary Material**

**Untreated HIV-1 infection and low CD4^+^ T cell counts and their effect on endemic HCoV (re)-infection**

Ferdyansyah Sechan, Anne W. M. van den Hurk, T. Sonia Boender, Maria Prins, Amy Matser, Margreet Bakker, Neeltje A. Kootstra, and Lia van der Hoek

**
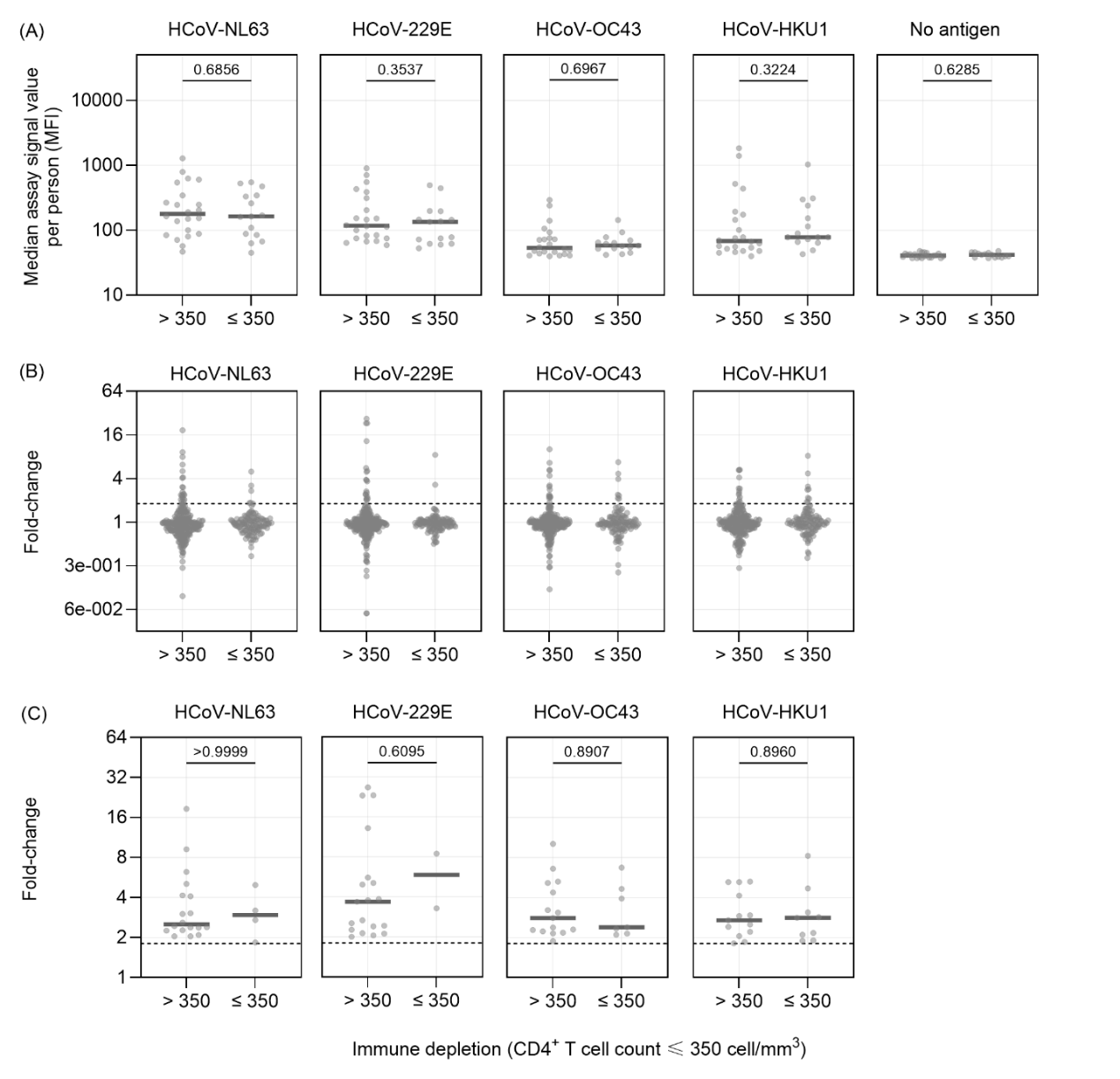
**

**S3 Fig. The distribution of anti-HCoV specific antibody level of PLWH with CD4^+^ T cell count above and below 350 cell/mm^3^, presented as the median antibody level per person (A), fold-change values at all data points (B), and all fold change-values ≥ 1.8 as the indication of infection (C).** Each dot in panel (A) represents one person and values are represented as median fluorescence intensity (MFI). Each dot in panel (B) and (C) represent one visit (data point) and values are represented as fold-change. Dashed black line at panel (B) and (C) represents the fold-change of 1.8 to indicate an infection by endemic HCoVs. Distribution of each dataset in panel (A) and (C) is depicted as median (solid grey line), and comparison in these panels was done using the Mann-Whitney U test.
